# Insights into the Transposable Mobilome of *Paracoccus* spp. (*Alphaproteobacteria*)

**DOI:** 10.1371/journal.pone.0032277

**Published:** 2012-02-16

**Authors:** Lukasz Dziewit, Jadwiga Baj, Magdalena Szuplewska, Anna Maj, Mateusz Tabin, Anna Czyzkowska, Grazyna Skrzypczyk, Marcin Adamczuk, Tomasz Sitarek, Piotr Stawinski, Agnieszka Tudek, Katarzyna Wanasz, Ewa Wardal, Ewa Piechucka, Dariusz Bartosik

**Affiliations:** Department of Bacterial Genetics, Faculty of Biology, Institute of Microbiology, University of Warsaw, Warsaw, Poland; Universite Libre de Bruxelles, Belgium

## Abstract

Several trap plasmids (enabling positive selection of transposition events) were used to identify a pool of functional transposable elements (TEs) residing in bacteria of the genus *Paracoccus* (*Alphaproteobacteria*). Complex analysis of 25 strains representing 20 species of this genus led to the capture and characterization of (i) 37 insertion sequences (ISs) representing 9 IS families (IS*3*, IS*5*, IS*6*, IS*21*, IS*66*, IS*256*, IS*1182*, IS*1380* and IS*1634*), (ii) a composite transposon Tn*6097* generated by two copies of the IS*Pfe2* (IS*1634* family) containing two predicted genetic modules, involved in the arginine deiminase pathway and daunorubicin/doxorubicin resistance, (iii) 3 non-composite transposons of the Tn*3* family, including Tn*5393* carrying streptomycin resistance and (iv) a transposable genomic island Tn*Ppa1* (45 kb). Some of the elements (e.g. Tn*5393*, Tn*6097* and ISs of the IS*903* group of the IS*5* family) were shown to contain strong promoters able to drive transcription of genes placed downstream of the target site of transposition. Through the application of trap plasmid pCM132TC, containing a promoterless tetracycline resistance reporter gene, we identified five ways in which transposition can supply promoters to transcriptionally silent genes. Besides highlighting the diversity and specific features of several TEs, the analyses performed in this study have provided novel and interesting information on (i) the dynamics of the process of transposition (e.g. the unusually high frequency of transposition of Tn*Ppa1*) and (ii) structural changes in DNA mediated by transposition (e.g. the generation of large deletions in the recipient molecule upon transposition of IS*Pve1* of the IS*21* family). We also demonstrated the great potential of TEs and transposition in the generation of diverse phenotypes as well as in the natural amplification and dissemination of genetic information (of adaptative value) by horizontal gene transfer, which is considered the driving force of bacterial evolution.

## Introduction

Transposable elements (TEs) are components of nearly all prokaryotic genomes. They are mobilized to change their location in DNA by the action of a transposase (TPase), an enzyme which catalyses transposition, i.e. recombination that does not require sequence homology between the TE and the target site. The presence and activity of TEs may lead to structural changes in both the size and composition of a genome. TEs can generate (i) various mutations, such as insertions, deletions, duplications, inversions and translocations of even large DNA fragments, and (ii) replicon fusions [1]. Some elements are able to activate silent, promoterless genes, when they are located in close proximity to the target site of transposition [2], [3]. Moreover, TEs can mobilize chromosomal genes for transposition, which results in shuffling of genetic information among various replicons present in a bacterial cell [4]. Thus, these highly recombinogenic elements may be considered as major architects responsible for shaping the structure of prokaryotic genomes. Their activity significantly enhances genome variability and consequently the adaptative and evolutionary capacities of their hosts. In addition, TEs very frequently occur on mobile plasmids and bacteriophages, which enhances the propagation of TEs by horizontal gene transfer (HGT) between various bacteria. This results in significant enrichment of the bacterial mobilome, i.e. a pool of mobile DNA involved in HGT, which is considered to be the driving force of bacterial evolution.

The simplest TEs are insertion sequences (ISs), which are highly abundant in bacteria. They carry only genetic information that is necessary and sufficient for transposition and its regulation. The majority of ISs are composed of a single open reading frame (ORF), encoding a transposase, flanked by two inverted repeat sequences (IRs) that are recognized by the TPase during transposition. In most cases, the transposition of ISs causes duplication of the target site, so that the inserted element is bordered by two short (2–15 bp) identical direct repeat sequences (DRs) [5].

ISs are also able to form more complex TEs such as (i) composite transposons consisting of random segments of genomic DNA (core region) bordered by a pair of ISs, or (ii) transposable modules (TMos) and ISCR elements composed of one IS copy, which can mobilize an adjacent DNA segment for transposition [3], [6]. ISs are also responsible for the mobility of non-autonomous TEs (i.e. not encoding their own transposase), such as (i) mobile insertion cassettes (MICs), identified in *Bacillus cereus* [7] and (ii) miniature inverted repeat transposable elements (MITEs), which may constitute up to 2% of a bacterial genome [8].

There is another widely distributed group of TEs called the non-composite transposons, which is divided into the Tn*3* and Tn*7* families. Besides a transposase, the Tn*3* elements contain a site-specific recombination module encoding a resolvase (an enzyme responsible for resolution of cointegrates, which are intermediate forms in replicative transposition), while the Tn*7* elements are known for their complex transposition machinery (they encode five transposition proteins) [9], [10]. These transposons often encode beneficial functions such as drug or heavy metal resistance, pathogenicity or the ability to utilize different carbon sources [11], [12].

Large-scale genome sequencing projects have led to an explosion in the number of annotated prokaryotic and eukaryotic TEs (for example, more than 3800 IS elements have now been isolated from over 295 prokaryotic species; ISfinder database [13]). However, in most cases their activity has not been experimentally confirmed.

In 2009 we initiated a project aimed at identifying functional transposable elements harbored by bacteria belonging to the genus *Paracoccus* (*Alphaproteobacteria*). This genus currently comprises 31 species, which have been isolated from many geographical locations and from different environments. *Paracoccus* spp. are physiologically among the most versatile bacteria, and are able to perform a number of different growth modes. Many are aerobic chemoorganoheterotrophs utilizing a wide variety of organic compounds, including potential pollutants like acetone, *N*,*N*-dimethylformamide and methylamine [14]. Some strains of *Paracoccus* spp. are facultative chemolithoautotrophs utilizing reduced sulfur compounds, molecular hydrogen and Fe(II) as energy sources [14], [15]. Because of their versatile metabolism, these bacteria can play an important role in the cycling of chemical elements in the environment. In addition, these physiological properties raise the possibility of employing paracocci in bioremediation systems, particularly since many species can use nitrate as an alternative electron acceptor.

Although there is no direct evidence linking the presence of TEs with important phenotypic traits of *Paracoccus* spp., it is tempting to speculate that their physiological heterogeneity might result from various transposition events.

In this study, TEs residing in strains representing 20 *Paracoccus* species were analyzed. For the identification of functional TEs we used trap plasmids, which are convenient tools enabling the direct identification of even phenotypically silent elements (reviewed by Solyga and Bartosik) [16]. In preliminary studies, TEs residing in several strains of four species were examined: *P. pantotrophus* [17], [18], *P. solventivorans* [19], *P. marcusii* [20], and *P. methylutens* [3]. This allowed identification of (i) twelve insertion sequences – IS*Pme2*, IS*Ppa2*, IS*Ppa3*, IS*Ppa4*, IS*Pso2*, IS*Pso3* (IS*5* family), IS*Ppa5* (IS*66* family), IS*Ppa1*, IS*Pso1* (IS*256* family) and IS*1247a*, IS*Pme1* (IS*1380* family), IS*Pmar4* (IS*As1* family) [3], [17], [19], [20], (ii) two closely related transposons of the Tn*3* family – cryptic Tn*3434* and streptomycin resistant Tn*5393* [17], (iii) a transposable genomic island Tn*Ppa1*, whose transposition is mediated by two copies of Tn*3434* [18], and (iv) a group of TMos, which are composed of a single copy of an IS*1380*-family element and adjacent fragments of genomic DNA of various lengths [3], [20]. Thus, through this complex analysis we have defined a transposable mobilome in these *Paracoccus* spp. strains. We also analyzed the structure, frequency of transposition, specific features and distribution of the captured TEs, which allowed us to draw more general conclusions concerning their evolutionary impact, as well as the direction and frequency of horizontal gene transfer in this group of bacteria.

## Materials and Methods

### Bacterial Strains and Culture Conditions

For identification of transposable elements rifampicin-resistant (Rif^r^) derivatives of the wild-type strains of *Paracoccus* spp. (listed in Table 1) were used. *Escherichia coli* TG1 [21] was used for plasmid construction. The majority of the strains were grown in lysogeny broth (LB) medium (Sigma) at 30°C (*Paracoccus* spp.) or 37°C (*E. coli*). *P. homiensis* DSM 17862 was cultivated in Marine Broth (Difco). Where necessary, the medium was supplemented with sucrose (10%) and with antibiotics at the following concentrations (µg·ml^−1^): kanamycin, 50; rifampicin, 50; tetracycline, 0.3–35 (depending on the strain).

**Table 1:** *Paracoccus* spp. strains used in this study.

*Paracoccus* spp. strain	References
*P. aestuarii* DSM 19484	[59]
*P. alcaliphilus* JCM 7364	[60]
*P. alkenifer* DSM 11593	[61]
*P. aminophilus* JCM 7686	[62]
*P. aminovorans* JCM 7685	[62]
*P. bengalensis* DSM 17099	[63]
*P. denitrificans* LMD 22.21	[64]
*P. ferrooxidans* NCCB 1300066	[15]
*P. haeundaensis* LMG P-21903	[65]
*P. halophilus* JCM 14014	[66]
*P. homiensis* DSM 17862	[67]
*P. kondratievae* NCIMB13773^T^	[68]
*P. koreensis* JCM 21670	[69]
*P. marcusii* DSM 11574	[70]
*P. marcusii* OS22	[71]
*P. methylutens* DM12	[72]
*P. pantotrophus* DSM 11072	[73]
*P. pantotrophus* DSM 11073	[73]
*P. pantotrophus* DSM 65	[74]
*P. pantotrophus* LMD 82.5	[64]
*P. seriniphilus* DSM 14827	[75]
*P. solventivorans* DSM 6637	[76]
*P. solventivorans* DSM 11592	[76]
*P. sulfuroxidans* JCM 14013	[77]
*P. thiocyanatus* JCM 20756	[78]
*P. versutus* UW400	[79]
*P. yeei* CCUG 32052	[80]
*P. yeei* CCUG 32053	[80]
*P. yeei* CCUG 46822	[80]
*P. zeaxanthinifaciens* ATCC21588^1^	[81]

### Plasmids Used in This Study

For the identification of TEs, the following trap plasmids were used: pMEC1 [17], pMAT1 [20], pMMB2 [18], pEBB10 and pCM132TC [3]. The shuttle *E. coli*-*Paracoccus* spp. trap plasmids pMEC1 and pMMB2 contain the *cI-tetA* cassette, composed of (i) a silent tetracycline resistance gene *tetA* under the control of the bacteriophage lambda pR promoter, and (ii) the gene encoding the lambda cI repressor. Inactivation of the repressor gene (e.g. through insertion of an IS), results in constitutive expression of tetracycline resistance [22]. The broad host range (BHR) trap plasmid pMAT1 or its Tc^r^ version pEBB10, contains the *sacB* gene of *Bacillus subtilis*, coding for levan sucrase – an enzyme that catalyzes sucrose hydrolysis and levan extension. The products of this reaction are toxic for gram-negative bacteria. Therefore, cells carrying the functional *sacB* gene are sucrose sensitive (Suc^s^) and their cultivation in medium containing sucrose results in cell lysis [23]. This allows direct selection of *sacB* mutants (Suc^r^) (e.g. carrying inserted TEs), whose growth is not affected under these conditions, thus enabling positive selection of transposition mutants. The BHR trap plasmid pCM132TC, contains a promoterless tetracycline resistance gene *tetA*. In this case, the transposition of TEs containing strong promoters upstream of the *tetA* gene can initiate its expression, resulting in tetracycline resistance in cells carrying such mutated plasmids [3]. Other plasmids, used for analysis of the frequency of transposition of Tn*Ppa1* were (i) pBBR1MCS-3 [24], (ii) pDG12 (*oriV* of pMAR4 of *P. marcusii* DSM 11574; *oriT* RK2; Km^r^), (iii) pDIY703 [25], (iv) pMAO-oriT (*oriV* RA3; *oriT* of RK2; Km^r^), (v) pMAO-MS (*oriV* RA3; *oriT* of pIGMS31 of *Klebsiella pneumoniae* 287-w; Km^r^) [26], and (vi) pMAO-RK (*oriV* RA3; *oriT* of pIGRK of *K. pneumoniae* 287-w; Km^r^) [26].

### Introduction of DNA into Bacterial Cells

DNA was introduced into *Paracoccus* spp. strains by triparental mating as previously described [27]. Chemical transformation of *E. coli* cells was performed according to the method of Kushner [28].

### DNA Manipulation and PCR Conditions

Plasmid DNA was isolated using a standard alkaline lysis procedure [29] and when required, purified by CsCl-ethidium bromide density gradient centrifugation. Total DNA from *Paracoccus* spp. was isolated by the procedure described by Chen and Kuo [30]. Common DNA manipulation methods were performed as described by Sambrook and Russell [31]. DNA amplification by PCR was performed in a Mastercycler (Eppendorf) using synthetic oligonucleotides (listed in Table S1), HiFi or Taq DNA polymerase (Qiagen; with supplied buffer), dNTPs and appropriate template DNAs. PCR products were analyzed by electrophoresis on 0.8% or 2% agarose gels and where necessary, purified using a Gel Out Kit (A&A Biotechnology).

### Identification and analysis of a pool of TEs

Trap plasmids were introduced into the Rif^r^ strains of *Paracoccus* spp. The overnight cultures of the transconjugants were spread on plates with solidified LB medium supplemented with tetracycline (in the case of pMEC1, pMMB2 or pCM132TC) or sucrose (pMAT1 or pEBB10). Appropriate dilutions of the cultures were also spread on LB medium in order to determine the frequency of transposition. For each strain, the plasmids of at least 100 Tc^r^ or Suc^r^ clones were analyzed. Three classes of plasmids were distinguished carrying (i) potential insertion sequences (inserts <3 kb), (ii) putative transposons (inserts >3 kb) or (iii) point mutations (replicons of the same size as the trap plasmid). The insertion sites of the elements were localized by performing PCRs with the trap plasmid insertion derivatives (as template DNA) and previously described sets of cassette-specific primers [17], [20]. Unique TEs identified in each strain (selected on the basis of restriction and DNA hybridization analyses) were further sequenced.

### Determination of the Genomic Location of Tn*6097* by IPCR

Templates for inverse PCR (IPCR) were prepared from total DNA of *Paracoccus ferrooxidans* NCCB 1300066 digested with restriction endonucleases NcoI or XhoI. The digested DNA preparations, purified using a Clean Up kit (A&A Biotechnology), were then self-ligated overnight at 16°C with T4 DNA ligase (Fermentas). The ligation mixture was used as the template for amplification by PCR of the DNA flanking Tn*6097* in the host genome. The synthetic oligonucleotides used in PCR were LIPCRPF2 and RIPCRPFE (Table S1).

### DNA-DNA Hybridization

Dot blot analysis was performed using a Bio-Dot apparatus (Bio-Rad) according the manufacturer's instructions. To determine copy number of TEs in parental strains Southern blotting was carried out as described by Sambrook and Russell [31]. Probe DNA fragments of the transposase gene were amplified by PCR using specific oligonucleotide primer pairs listed in Table S1. The fragments were gel-purified and labeled with digoxigenin (Roche). Hybridization and visualization of bound digoxigenin-labeled probes were carried out as recommended by the supplier (Roche). For each of the tested elements, a specific probe was hybridized with appropriately digested total DNA and plasmid DNA of the host strain. The restriction enzymes for DNA digestion were carefully selected for each of the tested elements to avoid multiple hybridization signals derived from a single copy of a given IS. The number of DNA bands hybridizing with the probe was therefore equivalent to the minimum number of copies of a given element within the genome.

### Localization of Promoters Enabling Activation of Transcriptionally Silent Genes

A pool of derivatives of the trap plasmid pCM132TC (containing inserted TEs able to drive transcription of a promoterless *tetA* gene in this vector) were analyzed to localize the TE-derived promoters. Deletion analysis was performed for two of the analyzed elements (Tn*5393* and Tn*6097*), by removal of different parts of the inserted TE by digestion with appropriate restriction enzymes, followed by religation and characterization of the resistance phenotype. The constructed mutants contained different parts of the 3′-end of the transposon, adjacent to the promoterless *tetA* gene. For the insertion sequences IS*Pam1*, IS*Pkr1* and IS*Paes3*, PCR was applied to amplify (i) the terminal 3′-end DNA fragments of the elements (identification of possible terminal, outwardly oriented promoters) and (ii) analogous fragments with an attached short DNA region of the trap plasmid, adjacent to the target site of transposition (identification of possible hybrid promoters). The amplified DNA fragments were cloned into pCM132TC (Tc^s^) upstream of the promoterless *tetA* gene or into pCM132 upstream of promoter-less *lacZ* reporter gene. The presence of a promoter (tested in *Paracoccus* spp. hosts) resulted in a Tc^r^ phenotype (pCM132TC) or expression of β-galactosidase (pCM132). β-galactosidase activity was measured as previously described [32]. The same strategy was used to localize promoters with a pCM132TC::pKLW1 co-integrate. Primers used for PCR amplification are listed in Table S1.

### DNA Sequencing, Sequence Analyses and Annotation

Nucleotide sequences of TEs were determined using a dye terminator sequencing kit and an automatic sequencer (ABI 377 Perkin Elmer). A combination of vector-derived primers and primer walking was used to obtain the entire nucleotide sequences. Similarity searches were performed using the ISfinder [33] and BLAST programs [34] provided by the National Center for Biotechnology Information (http://www.ncbi.nlm.nih.gov/). G+C plots were created using the program Artemis [35] with a window setting of 70 nucleotides. The numbering of Tn*6097* and Tn*6122* was assigned by the Tn Number Registry website (UCL Eastman Dental Institute; http://www.ucl.ac.uk/eastman/tn/) according to the generally accepted nomenclature [36].

### Nucleotide Sequence Accession Numbers

The nucleotide sequences of the elements identified in this study have been submitted to the ISfinder and GenBank databases. Accession numbers of the sequences are given in Table 2.

**Table 2:** Transposable elements of *Paracoccus* spp. identified in this study.

TE	TE family/group	Length (bp)	IR (bp)^a^	DR (bp)	Acc. no. or reference	Host strain (copy number; location^b^)
**IS** ***1247***	IS*1380*	1672	16/13	5	[82]	*P. halophilus* JCM 14014 (2; 1 in pHAL1)
**IS** ***1248***	IS*5*/IS*427*	832	14/13	2	[83]	*P. pantotrophus* DSM 11073 (2)
**IS** ***1248f***	IS*5*/IS*427*	832	14/13	2	GQ871939	*P. pantotrophus* LMD 82.5 (6)
**IS** ***Paes1***	IS*5*/IS*427*	851	18/16	2	GU826197	*P. aestuarii* DSM 19484 (6)
**IS** ***Paes2***	IS*5*/IS*427*	841	21/18	2	GU826198	*P. aestuarii* DSM 19484 (2)
**IS** ***Paes3***	IS*256*	1393	39/27	8	GU826199	*P. aestuarii* DSM 19484 (2)
**IS** ***Pak1***	IS*5*/IS*427*	853	15/13	2	GQ871938	*P. alkenifer* DSM 11593 (1)
**IS** ***Pam1***	IS*5*/IS*903*	1050	15/15	9	GQ468940	*P. aminophilus* JCM 7686 (3)
**IS** ***Pam2***	IS*5*/IS*903*	1054	19/18	9	GQ468941	*P. aminophilus* JCM 7686 (2)
**IS** ***Pam3***	IS*3*/IS*407*	1197	39/30	4	[32]	*P. aminophilus* JCM 7686 (1 in pAMI2)
**IS** ***Pam4***	IS*5*/IS*427*	865	15/13	2	[32]	*P. aminophilus* JCM 7686 (7; 1 in pAMI2)
**IS** ***Pbe1***	IS*3*/IS*407*	1198	12/12	2	GQ871936	*P. bengalensis* DSM 17099 (6)
**IS** ***Pbe2***	IS*5*/IS*427*	851	18/15	2	HQ384167	*P. bengalensis* DSM 17099 (4)
**IS** ***Pfe1***	IS*3*/IS*407*	1248	15/15	4	HQ384168	*P. ferrooxidans* NCCB 1300066 (7)
**IS** ***Pfe2***	IS*1634*	1884	13/13	6	HQ384169	*P. ferrooxidans* NCCB 1300066 (2)
**IS** ***Pha1***	IS*5*/IS*427*	842	13/12	4	GU826204	*P. halophilus* JCM 14014 (6)
**IS** ***Pha2***	IS*5*/IS*5*	1387	16/15	4	GU826205	*P. halophilus* JCM 14014 (1 in pHAL1)
**IS** ***Phae1***	IS*5*/IS*5*	1485	16/13	6	GQ868754	*P. haeundaensis* LMG P-21903 (2)
**S** ***Pko1***	IS*5*/IS*903*	1050	18/18	9	GQ868751	*P. kondratievae* NCIMB 13773^T^ (4 in pKON1)
**IS** ***Pko1a***	IS*5*/IS*903*	1050	18/18	9	GU826201	*P. koreensis* JCM 21670 (2)
**IS** ***Pkr1***	IS*21*	2469	34/26	8	GU826200	*P. koreensis* JCM 21670 (2)
**IS** ***Plc1***	IS*5*/IS*903*	1050	27/23	9	GQ864160	*P. alcaliphilus* JCM 7364 (8)
**IS** ***Pmar1***	IS*6*	821	21/18	9	GU997095	*P. marcusii* DSM 11574 (2)
**IS** ***Pmar2***	IS*3*/IS*407*	1269	25/18	4	GU997096	*P. marcusii* DSM 11574 (1)
**IS** ***Pmar3***	IS*5*/IS*5*	1282	18/14	4	GU826203	*P. marcusii* OS22 (2; 1 in pMOS8)
**IS** ***Ppa2***	IS*5*/IS*427*	832	14/13	2	[17]	*P. methylutens* DM12 (3)
						*P. ferrooxidans* NCCB 1300066 (2)
**IS** ***Ppa3a***	IS*5*/IS*903*	1054	21/19	9	GQ864161	*P. alcaliphilus* JCM 7364 (6)
**IS** ***Ppa5a***	IS*66*	2829	22/20	8	GQ871937	*P. bengalensis* DSM 17099 (4)
**IS** ***Ppa6***	IS*5*/IS*427*	850	15/12	2	EU909900	*P. pantotrophus* DSM 11073 (8)
**IS** ***Ppa7***	IS*66*	2731	35/26	8	EU909901	*P. pantotrophus* DSM 65 (7)
**IS** ***Ppa8***	IS*5*/IS*903*	1050	17/16	9	EU909902	*P. pantotrophus* DSM 65 (9)
						*P. pantotrophus* DSM 11073 (3)
**IS** ***Ppa9***	IS*6*	812	15/15	8	EU909903	*P. pantotrophus* DSM 65 (1)
**IS** ***Pse1***	IS*1182*	1653	16/16	4	GQ868753	*P. seriniphilus* DSM 14827 (1)
**IS** ***Pth1***	IS*5*/IS*903*	1054	18/15	9	GQ868752	*P. thiocyanatus* JCM 20756 (2)
**IS** ***Pve1***	IS*21*	2503	19/13	8	HQ384165	*P. versutus* UW400 (1)
**IS** ***Pve1*** **a**	IS*21*	2503	29/19	7	HQ384166	*P. bengalensis* DSM 17099 (6)
**IS** ***Pze1***	IS*5*/IS*427*	851	18/13	2	GU826202	*P. zeaxanthinifaciens* ATCC 21588^T^ (6)
**Tn** ***3434*** **a**	Tn*3*	3695	38/38	6	[25]	*P. aminophilus* JCM 7686 (4; 1 in pAMI1, 1 in pAMI7 and 2 in pAMI8)
**Tn** ***5393***	Tn*3*	5470	81/77	6	[17]	*P. pantotrophus* LMD 82.5 (1)
**Tn** ***6097***		17,759	13/13	6	JN122276	*P. ferrooxidans* NCCB 1300066 (1)
**Tn** ***6122***	Tn*3*	3792	39/39	5	JN127372	*P. halophilus* JCM 14014 (1 in pHAL1)
**Tn** ***Ppa1***	Tn*3*	44,386	35/35	5	[18]	*P. pantotrophus* DSM 11072 (1)

a The length of the IRs/the number of identical residues.

b Location in plasmids, if applicable.

## Results

### Identification of a pool of functional TEs in *Paracoccus* spp

To identify functional TEs of *Paracoccus* spp. we used mobilizable trap plasmids pMEC1, pMMB2, pMAT1, pEBB10 and pCM132TC carrying three types of cassette enabling positive selection of transposition events (see Materials and Methods). The plasmids pMEC1, pMMB2, pMAT1 and pEBB10 (containing the *cI-tetA* or *sacB* cassettes) enable the capture of all types of TEs, irrespective of their structure, specific features and genetic load. In contrast, pCM132TC only permits the identification of elements containing strong outwardly oriented promoters or those able to generate hybrid promoters, which drive the transcription of nearby genes.

**Figure 1:**
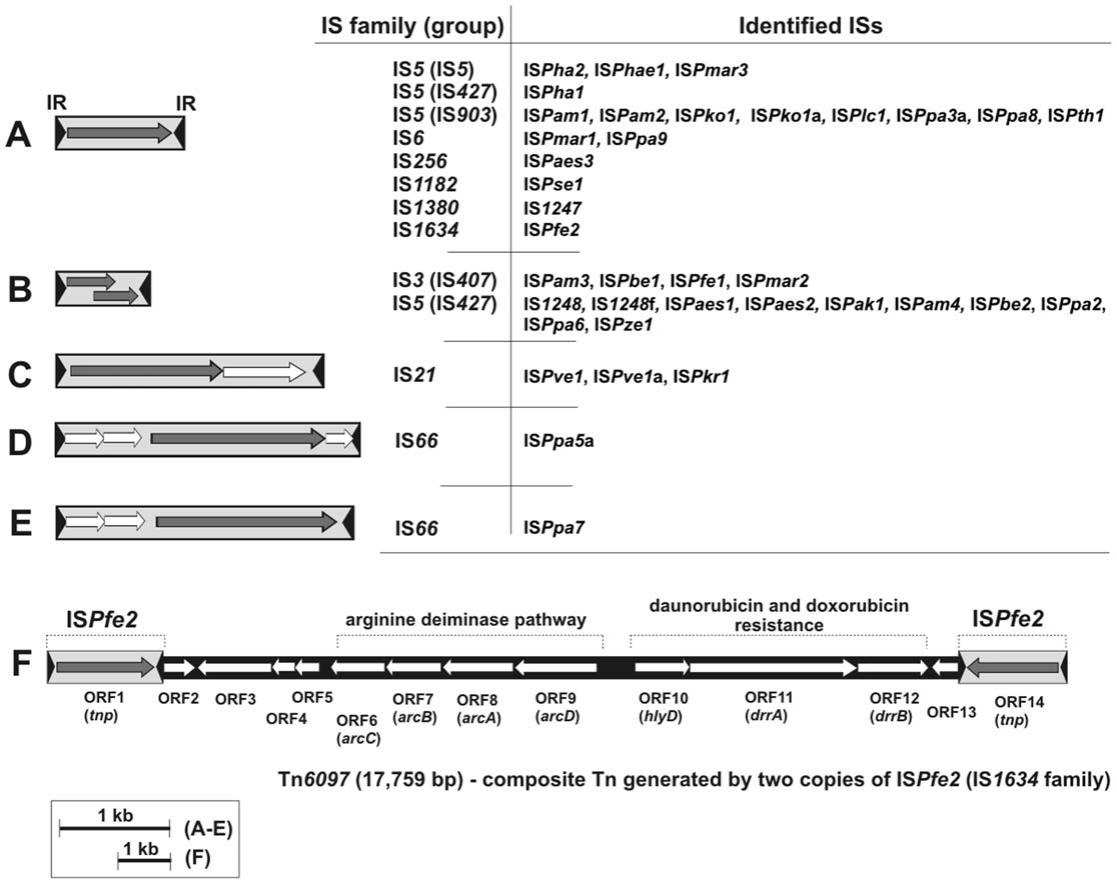
Genetic organization of the insertion sequences (A, B, C, D, E) and composite transposon Tn*6097* (F) of *Paracoccus* spp. identified in this study. The families and names of the identified ISs are shown on the panel. Inverted repeats (IRL – left IR; IRR – right IR) flanking ISs are marked by black arrowheads. Predicted coding regions are represented by arrows indicating the direction of transcription. Gray arrows indicate TPase genes and white arrows indicate additional ORFs present within the ISs and within the core of Tn*6097*. Two of the ORFs of Tn*6097* (ORF2 and ORF13) are truncated (the disruptions are most probably remnants of the ancient IS*Pfe2* transposition events that led to the formation of Tn*6097* in its native host). The predicted genetic modules of the transposon are indicated.

**Figure 2:**
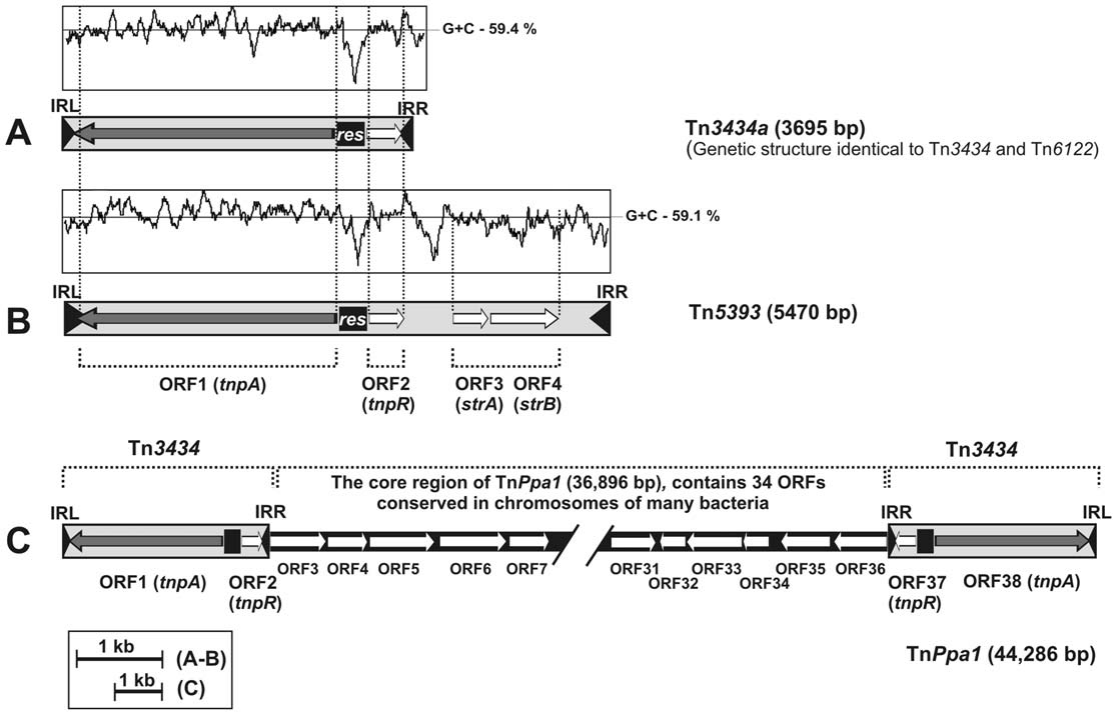
Genetic organization of the Tn*3* family transposons captured in *Paracoccus* spp. using trap plasmids. The cryptic transposon Tn*3434*a (A), Tn*5393* carrying streptomycin resistance genes (B) and the composite element Tn*Ppa1* flanked by two identical copies of Tn*3434* (C). Predicted coding regions are represented by arrows indicating the direction of transcription. Gray arrows indicate TPase genes (*tnpA*) and additional genes (including resolvase genes – *tnpR*) are indicated by white arrows. Inverted repeats (IRs) flanking transposons are marked by black arrowheads (IRL – left IR; IRR – right IR) and *res* sites are shown by black squares. Plots of the G+C content of the Tn*3434*a and Tn*5393* sequences are shown in panels A and B (the average value is given on the right).

The trap plasmids were introduced by conjugation into 25 strains of *Paracoccus* spp. and pools of clones carrying mutated plasmids were obtained and analyzed as described in Materials and Methods. Using this strategy, we captured (i) 37 insertion sequences, (ii) one composite transposon and (iii) 3 non-composite transposons belonging to the Tn*3* family (Figures 1 and 2). TEs were identified in the majority of tested strains, with the exception of *P. aminovorans* JCM 7685, *P. homiensis* DSM 17862, *P. sulfuroxidans* JCM 14013 and *P. yeei* (strains CCUG 46822 and CCUG 32052).

### Insertion sequences

The nucleotide sequences of the captured ISs were subjected to detailed analysis. All contained a transposase gene(s) and terminally placed inverted repeat sequences (IRs). Moreover, all were able to generate DRs at the target site of transposition (Table 2). Since they were “typical” ISs, their nucleotide sequences as well as amino acid (aa) sequences of their predicted transposases (TPases), were subjected to comparative analyses using the ISfinder (gathers the nucleotide sequences of well defined ISs) and NCBI databases (BLAST program).

Most bacterial transposases possess three domains (designated N2, N3 and C1), which contain three conserved residues (two aspartate and one glutamate) constituting the DDE motif (so-called catalytic triad), that is highly conserved in the majority of bacterial TPases [5] (see Figure S1). The spacing between these residues as well as the presence of other conserved residues within the domains varies between different IS families or groups [5]. Since the number of described ISs has increased significantly in recent years (800 known ISs in 2002 and >3800 in 2011), we performed a multiple alignment of the amino acid sequences of the transposases of ISs present in the ISfinder database with all the *Paracoccus* spp. ISs identified in this study. Analogous analysis was also used to compare the nucleotide sequences of the terminal IRs of the ISs (Figure S1).

Preliminary analysis revealed that the vast majority (32) of the identified ISs were novel elements. In five cases, we captured ISs that were highly related to known elements (since their transposases showed >95% identity, at the amino acid level, they were considered different isoforms of the same element) – IS*1248*f, IS*Pko1*a, IS*Ppa3*a, IS*Ppa5*a and IS*Pve1*a. In only four cases we found completely identical elements residing in different bacterial strains – IS*Ppa2*, IS*1247*, IS*1248* and IS*Ppa8*. The identified ISs and their characteristics are presented in Table 2.

Based on the results of these comparative analyses, the ISs of *Paracoccus* spp. could be classified into the following families: (i) IS*3* family (IS*407* group) – 4 captured elements, (ii) IS*5* family (IS*5*, IS*427* and IS*903* groups) – 22 elements, (iii) IS*6* family – 2, (iv) IS*21* family – 3, (v) IS*66* family – 2, (vi) IS*256* family – 1, (vii) IS*1182* family – 1, (viii) IS*1380* family – 1, and (ix) IS*1634* family – 1.

As shown in Figure 1A, the majority of the ISs contain a single ORF encoding a transposase. However, some of them (belonging to the IS*407* group of the IS3 family and IS*427* group of the IS*5* family) carry two overlapping ORFs and possess a conserved frameshift motif (Figure 1B), which is likely to promote the generation of a fusion protein (ORF1+ORF2) as a result of programmed translational frameshifting [37]. In each case, only the predicted fusion protein contains the complete DDE motif characteristic of functional transposases. Since the generation of fusion proteins is not a frequent phenomenon (as shown for IS*1* of *E. coli*) [37], it is apparent that ribosomal frameshifting may participate in negative regulation of the transposition of these elements. So far, the production of such a transframe protein has been reported exclusively for members of the IS*1* and IS*3* families [37], [38].

Interestingly, we found that one of the members of the IS*427* group of the IS*5* family (IS*Pha1* of *P. halophilus* JCM 14014) is a natural point mutant, because, in contrast to other elements of this group, it carries a single large ORF encoding a functional DDE transposase.

The third class of the identified ISs consists of elements that contain additional ORF(s) besides the transposase gene(s). In most cases, the specific role of the predicted proteins encoded by these ORFs is unknown, although it is highly probable that they are involved in the regulation of the process of transposition. Such multi-ORF structures are typical for members of the IS*21* and IS*66* families (Figure 1CDE).

We also performed a detailed inspection of the nucleotide sequences of the captured ISs in order to predict any DNA sequences that may constitute a site of interaction with host-encoded factors. This analysis revealed that the majority of the elements of the IS*427* group of the IS*5* family (8 ISs) contain, within their left IR (IRL; located at the 5′ end of the element), a sequence partially matching the consensus sequence of the integration host factor (IHF) binding site (5′- WATCAANNNNTTR -3′) [39]. IHF is a DNA-bending protein, which influences genome architecture. It may also act as a positive factor in the transposition of some TEs, e.g. IS*10* [40]. It is probable that it also modulates the transposition of elements of the IS*427* group.

### Composite transposon Tn*6097*

The only composite transposon identified in this study was a novel element, designated Tn*6097* (17,759 bp), which was captured in *P. ferrooxidans* NCCB 1300066 using the trap plasmid pCM132TC. A summary of the predicted ORFs of Tn*6097*, including their position, the size of the encoded proteins, and their closest homologs is presented in Table S2.

Transposition of Tn*6097* into the selective cassette of pCM132TC generated 6-bp DRs. This transposon contains two identical, convergently oriented copies of IS*Pfe2* (1884 bp), that are responsible for its mobility (Figure 1F). Using trap plasmid pMAT1, we also demonstrated the transposition of a single copy of IS*Pfe2*, which generated DRs of the same length as Tn*6097* upon insertion into the *sacB* gene. IS*Pfe2* has been classified within the IS*1634* family. It is the only member of this family identified so far in *Paracoccus* spp. The TPase of IS*Pfe2* and that encoded by the most closely related element, IS*Thsp7* of *Thiomonas* sp. 3As (accession no FP475956) (*Betaproteobacteria*), share 51% aa sequence identity.

The two copies of IS*Pfe2* flank the large core region of Tn*6097*, comprised of 13,991 bp. The average G+C content of this region is 63.6 mol%, which is lower than that of the total DNA of the native host *P. ferrooxidans* NCCB 1300066 (67 mol%). The core region contains 12 ORFs whose putative products show similarity to proteins conserved in many bacteria, including some involved in the metabolism of nitrogen compounds and resistance to chemotherapeutics. Based on the predicted functions of the ORFs, they may be divided into three clusters, which can also be differentiated by the G+C content of their nucleotide sequences (data not shown).

The first cluster contains four ORFs (ORF2-ORF5) (Figure 1F) encoding putative proteins involved in nitrogen metabolism: (i) a FtrB/NarB-like regulator of transcription with significant aa sequence similarity (51%) to a positive transcription regulator of a respiratory nitrate reductase gene from a strain of *P. pantotrophus* [41], (ii) proteins involved in the response to NO [42] and (iii) a protein responsible for the transformation of *5*-formyltetrahydrofolate and ATP into *5,10*-methenyltetrahydrofolate, ADP and phosphate, which results in the creation of carbon-nitrogen bonds due to the cyclo-ligase activity [43].

The second cluster of Tn*6097* contains four ORFs (ORF6-ORF9) (Figure 1F) with significant similarity to *arcABCD* genes, encoding the arginine deiminase pathway. This pathway permits bacteria to grow anaerobically with arginine as the substrate by catalyzing its conversion to ornithine, CO_2_, and NH_3_, with the generation of ATP [44]. The *arcABCD* genes encode (i) arginine/ornithine antiporter (ArcD), (ii) arginine deiminase (ArcA), (iii) ornithine carbamoyltransferase (ArcB) and (iv) carbamate kinase (ArcC), respectively. The *arcABCD* operon of Tn*6097* exhibit the highest nucleotide sequence similarity (76%) and synteny to homologous genes in plasmid pOANT03 of *Ochrobactrum anthropi* ATCC 49188 (accession no. NC_009671).

The third putative genetic module of Tn*6097* consists of three ORFs (ORF10-ORF12) (Figure 1F) encoding (i) a putative secretion protein of the HlyD family, which groups efflux pumps involved in resistance to various antibiotics and heavy metals, and (ii) two putative ABC-type transporters with similarities to ATP-binding proteins DrrA and DrrB, which are components of a bacterial exporter complex. DrrA and DrrB confer resistance to daunorubicin and doxorubicin (chemotherapeutics of the anthracycline family) [45]. The antibiotics are active exclusively against gram-positive bacteria (they do not enter the cells of gram-negative strains). Tn*6097* also carries a truncated ORF (ORF13; lacking its *N*-terminal coding part) adjacent to the right-hand copy of IS*Pfe2*, which encodes a putative NAD-dependent epimerase/dehydratase that is most similar (85% aa identity) to a protein of *P. denitrificans* PD1222 (Figure 1F).

DNA hybridization analysis revealed that Tn*6097* is present in one copy in the *P. ferrooxidans* NCCB 1300066 genome (data not shown). Using inverse PCR (IPCR) we determined the nucleotide sequence of its original target site in the host genome (see Materials and Methods for details). This revealed that Tn*6097* is located within a gene (disrupted upon transposition), whose predicted product shows similarity to a number of bacterial proteases. The highest identity (67%) was observed with a *htpX* gene of *Polymorphum gilvum* SL003B-26A1 (accession no YP_004302375). The transposon was flanked by 6-bp long DRs (5′-GGCTCG-3′), confirming that it had been incorporated into the host genome by means of transposition.

### Transposons of the Tn*3* family

A common feature of all captured non-composite transposons (three elements) was the presence of two putative genetic modules (responsible for transposition and resolution of cointegrates resulting from replicative transposition), which are highly conserved in members of the Tn*3* family of transposons. Two of the identified transposons (Tn*6122* and Tn*3434*a) were identified as cryptic elements, solely encoding genetic information required for transposition (Figure 2 and Table 2).

Tn*6122* (3792 bp) was captured in *P. halophilus* JCM 14014 using the trap plasmid pMEC1. This element is composed of (i) identical 39-bp IRs, (ii–iii) two divergently oriented ORFs encoding a large transposase protein (TnpA; 967 aa) and a resolvase (TnpR) as well as (iv) an AT-rich putative recombination site (*res*) separating the ORFs (this region is involved in cointegrate resolution and regulation of expression of the *tnpA* and *tnpR* genes) [11]. Comparative sequence analysis revealed that homologous cryptic transposons occur within the genomes of three strains belonging to the *Alphaproteobacteria*: (i) *Sulfitobacter* sp. NAS-14.1, (ii) *Ruegeria* sp. PR1b and (iii) *Roseovarius* sp. TM1035. These transposons encode highly related transposases and resolvases, and are bordered by IRs identical to those of Tn*6122* (data not shown).

The second trapped cryptic transposon, Tn*3434*a (3695 bp), identified using pEBB10 in *P. aminophilus* JCM 7686, is an isoform of the transposon Tn*3434*, that we trapped previously in *P. pantotrophus* DSM 11072 [17]. The newly identified isoform shares 98% nucleotide sequence identity with Tn*3434* (36 mismatches). The resolvases encoded by these elements are 100% identical, while their transposases differ in 30 aa (data not shown).

Tn*3434*a is also related to Tn*6122*. As shown in Figure 2, both elements have an identical genetic organization. They encode highly related transposases (73% aa identity), but their resolvases are more divergent (28% identity).

In a previous study using pMEC1, we showed that *P. pantotrophus* LMD 82.5 contains a non-composite transposon Tn*5393*, which is another member of the Tn*3* family [17]. This element, in contrast to the closely related Tn*3434*a and Tn*6122*, carries two streptomycin resistance genes (*strA* and *strB*) placed downstream of the *tnpR* gene (Figure 2). Interestingly, in the present study we observed transposition of this element (in the strain LMD 82.5) into the selection cassette of the trap plasmid pCM132TC, which revealed that this transposon can drive the transcription of genes placed downstream of the target site of transposition.

All three trapped non-composite transposons contained related IRs. The IRs of Tn*3434*a and Tn*6122* differ slightly in length (38 bp and 39 bp, respectively) and their sequence identity is only 50% (14 mismatches). In contrast, the IRs of Tn*5393* are much longer (81 bp with 4 mismatches between IRL and IRR), but their termini show significant similarity to those of Tn*3434*a, Tn*6122* and other members of the Tn*3* family (Figure S1). Despite their differences, the transposition of all these elements produced a 6-bp duplication of an AT-rich target sequence (Table 2).

During analysis carried out in *P. pantotrophus* DSM 11072, we captured another Tn*3* element, Tn*Ppa1* (driven by Tn*3434*), which was previously identified in this strain [18]. However, further analyses performed in the present study revealed an unusual feature of this element, which is described below.

### Specific features of the identified TEs

Analyses performed using trap plasmids not only enabled the capture of many functional TEs but also identified specific features of several elements, including their ability to (i) activate transcriptionally silent genes, (ii) generate deletions within the target sites of transposition and (iii) transpose at unusually high frequency.

### TEs enabling transcription of downstream genes

Using the trap plasmid pCM132TC (contains a promoterless tetracycline resistance gene as the selection cassette) we identified several TEs that are able to drive the transcription of promoterless genes placed downstream of the target site of transposition. The majority of the elements identified in this way were insertion sequences from four different groups: (i) the IS*903* group of the IS*5* family (IS*Pam1* and IS*Ppa8*), (ii) the IS*407* group of the IS*3* family (IS*Pam3* and IS*Pfe1*), (iii) the IS*21* family (IS*Pkr1*, IS*Pve1*) and (iv) the IS*256* family (IS*Paes3*). We also captured two transposons: (i) Tn*5393* of the Tn*3* family and (ii) the novel composite transposon Tn*6097* bordered by two copies of IS*Pfe2* (IS*1634* family).

**Figure 3:**
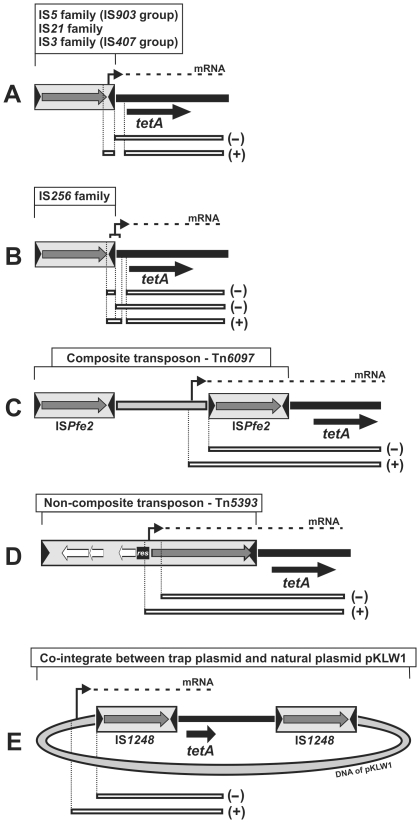
Schematic overview of five ways in which transposition can deliver promoters to the transcriptionally silent tetA (tetracycline resistance) gene of the trap plasmid pCM132TC. The location of promoters in the plasmids pCM132TC::TE, conferring a Tc^r^ phenotype, are appropriately indicated: an outwardly oriented promoter in the terminal parts of a TE (A), a hybrid promoter composed of a −35 hexamer (delivered by the TE) and a −10 hexamer located in close proximity to the target site of transposition (B), the promoter of a TPase gene (C), a promoter present in the core region of a composite transposon (D), and a promoter derived from another plasmid delivered by the generation of transient co-integrates resulting from replicative transposition (E). DNA fragments used in the localization of the promoters are shown as open thin boxes below each panel. The activity of promoters (tested in *Paracoccus* spp.) accompanying the presence of the DNA fragments is indicated on the right: (+) promoter activity, (−) lack of promoter activity.

In order to localize the promoters responsible for the resistance phenotype, we analyzed the TEs trapped within pCM132TC in the Tc^r^ clones, as described in Materials and Methods. The results of these experiments (summarized in Figure 3) revealed that there are several possible ways in which transposition can deliver promoters to transcriptionally silent genes. The transcription of nearby genes can be driven from (i) outwardly oriented promoters located in the terminal parts of the ISs – this is the case for all tested members of the IS*5* (IS*903* group), IS*3* (IS*407* group) and IS*21* families (Figure 3A), (ii) a hybrid promoter, most probably composed of a −35 hexamer present in the terminal part of the TE and a −10 hexamer located in close proximity to the target site of transposition (suggested for the IS*256* member) (Figure 3B), (iii) the promoter of the transposase gene (Tn*5393* of the Tn*3* family) (Figure 3C), or (iv) a promoter present in a core region of the composite transposon Tn*6097* (Figure 3D).

An unexpected phenomenon was observed upon selection of Tc^r^ clones in *P. pantotrophus* DSM 11073 containing pCM132TC. All of the obtained Tc^r^ mutants lacked the autonomous form of the trap plasmid, and DNA hybridization analysis revealed that in every case, the plasmid was present as part of a co-integrate generated between pCM132TC and plasmid pKLW1 (approx. 100 kb) which naturally occurs in strain DSM 11073.

DNA sequencing revealed that the recombinational event that led to the formation of these co-integrates occurred within the selection cassette of pCM132TC (upstream of the *tetA* gene). The inserted trap plasmid was bordered by two copies of the insertion sequence IS*1248* (IS*427* group of the IS*5* family), which strongly suggests that co-integrates were generated upon replicative transposition of this IS (Figure 3E).

The co-integrates therefore represent intermediate forms of transposition, which should be resolved by homologous recombination into individual, separate plasmids. After approximately 100 generations of growth of the strains under non-selective conditions we observed (by DNA electrophoresis; data not shown) the appearance of a DNA band corresponding to the resolved trap plasmid. In contrast, we were never able to detect individual replicons when plasmid DNA was isolated from bacteria grown in medium supplemented with tetracycline, which strongly suggests that this antibiotic provides selection for the maintenance of the co-integrates.

DNA sequencing of the selection cassette of the resolved trap plasmid confirmed the presence of an inserted copy of IS*1248*. When introduced into the strain DSM 11073, this plasmid (pCM132TC::IS*1248*), did not confer the Tc^r^ phenotype, which precludes the possibility that a promoter of the IS may drive transcription of a nearby gene.

These observations indicate that expression of the *tetA* gene in the co-integrate is driven by a promoter located within pKLW1. This conclusion is supported by the DNA sequence of regions flanking the original insertion site of IS*1248* within pKLW1 and the detection of activity of the upstream promoter (Figure 3E).

### TEs generating deletions

The transposition of the vast majority of TEs identified in this study resulted in the simple insertion of the elements into the trap plasmids and the generation of DRs. However, we found that the insertion of two ISs (IS*Pmar2* of *P. marcusii* DSM 11574 and IS*Pve1* of *P. versutus* UW400) into selection cassettes could be accompanied by deletions at the transposition target site.

In the case of IS*Pmar2* (IS*407* group of the IS*3* family) we identified only one deletion mutant (out of five analyzed pMEC1-derivatives containing inserted IS*Pmar2*). The deletion (15 bp) occurred at the 3′-end of the inserted IS, and therefore the intact element was not bordered by the DRs, in contrast to the other clones tested.

More interesting was the case of IS*Pve1* (IS*21* family), which frequently generated much larger deletions within the selection cassettes of various trap plasmids. Initial analysis of a pool of Tc^r^ clones of strain UW400 (obtained with pMEC1) revealed the presence of two types of pMEC1 mutants representing either (i) simple insertion of IS*Pve1* (7% of Tc^r^ clones), or (ii) insertion of IS*Pve1* associated with deletions within the trap plasmid (16%). The remaining Tc^r^ clones did not contain any TEs.

**Figure 4:**
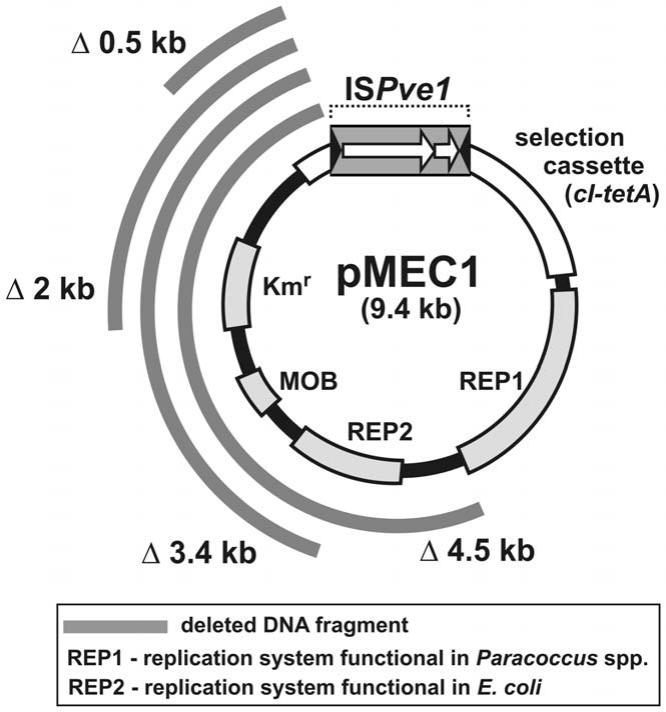
Deletions within the trap plasmid pMEC1 generated in *P. versutus* UW400 upon transposition of IS*Pve1*. The range of the deletion within four individual derivatives of pMEC1 is shown by curved gray lines.

In the case of simple insertions, transposition of IS*Pve1* resulted in the generation of 8-bp long DRs. Moreover, in both the insertion and deletion mutants, the element was always placed in the same orientation within the *cI* gene of pMEC1 (Figure 4). We further analyzed the IS*Pve1* insertion sites that underwent structural alterations and found that the deletions were always unidirectional, and they comprised different segments of the trap plasmid adjacent to the 5′ end of the IS (Figure 4). The deletions ranged in size from 0.5 to 4.5 kb. (Figure 4). Analogous deletions associated with the transposition of IS*Pve1* were also observed with other trap plasmids (e.g. pMMB2 and pMAT1) and in each case, the location of the replication system was the factor limiting the range of deletions generated within the plasmid (Figure 4 and data not shown).

### Frequently transposing TEs

In most cases, the frequency of transposition of the captured TEs ranged from 10^−8^ to 10^−6^ depending on the element and the bacterial strain (see Table S3). The lowest frequency (10^−10^) was observed for IS*Paes3 of P. aestuarii* DSM 19484. In contrast, the element most dynamic in transposition was Tn*5393* (*P. pantotrophus* LMD 82.5), which transposed into the selection cassette of the trap plasmid pCM132TC at a very high frequency (10^−3^). This is in agreement with our previous observations performed in the strain LMD 82.5 with pMEC1 [17].

An interesting phenomenon was observed after conjugal transfer of the trap plasmid pMEC1 into *P. pantotrophus* DSM 11072. Surprisingly, in this case, the vast majority of the transconjugants (92%) did not contain pMEC1 (9.4 kb) but they carried a very large replicon (>50 kb), that was not originally present in DSM 11072. The remaining transconjugants (8%) contained intact pMEC1. DNA hybridization followed by DNA sequencing revealed that the large nascent plasmids are in fact insertion mutants of pMEC1. They were generated by transposition into the trap plasmid of a large previously described composite transposon Tn*Ppa1* (44,286 bp) [18]. This transposon (considered to represent a transposable genomic island) is composed of two divergently oriented copies of the Tn*3* family transposon Tn*3434*, which border the core region of the element containing a set of genes conserved in the chromosomes of many bacteria (Figure 2C). The core region contains a large set of putative genes, whose products show similarity to enzymes involved in central intermediary metabolism (e.g. tricarboxylic acid cycle or 2-methylcitrate cycle), transporters, transcriptional regulators and conserved proteins of unknown function [18].

Tn*Ppa1* has previously been captured in strain DSM 11072R using pMMB2, although its transposition into the selection cassette of the trap plasmid was a relatively rare event (10^−6^) [18]. In the present study, we observed massive transposition of Tn*Ppa1* into pMEC1. Intriguingly, all the transposon insertions were detected in randomly tested Km^r^ transconjugants, i.e. without applying positive (tetracycline) selection for the identification of transposition events. DNA sequencing of several insertion derivatives of pMEC1 confirmed that transposition of Tn*Ppa1* had targeted random parts of the trap plasmid. In each case the inserted element was bordered by DRs, of a length typical for other members of the Tn*3* family (5 bp) (data not shown).

We also tested whether Tn*Ppa1* is able to transpose as frequently into other replicons. For this purpose, several mobilizable plasmids were transferred into strain DSM 11072R. The plasmids contained different replication systems derived from (A) three broad host range replicons – (i) RA3 (pMAO-oriT, pMAO-MS and pMAO-RK which contain different systems enabling mobilization for conjugal transfer), (ii) RK2 (pCM132TC) and (iii) pBBR1 (pMAT1, pBBR1MCS-3), as well as from (B) three plasmids originating from *Paracoccus* spp. – (i) pMAR4 of *P. marcusii* DSM 11574 (pDG12), (ii) pAMI7 of *P. aminophilus* JCM 7686 (pDIY703) and (iii) pTAV1 of *P. versutus* UW1 (pMMB2).

Analysis of the plasmid content of the obtained transconiugants revealed massive transposition of Tn*Ppa1* into plasmids containing replication systems of pMAR4 and pAMI7 as well as into all derivatives of RA3 (20–95% of tested clones carried Tn*Ppa1*, depending on the plasmid used), but not into the other tested plasmids (data not shown). This strongly suggested that the observed transposition phenomenon is dependent on the nature of the incoming replicon.

### Copy number, location and distribution of TEs in the genomes of *Paracoccus* spp

Southern blotting and DNA hybridization analysis were performed to examine the copy number and genomic location (plasmid/chromosome) of the identified TEs in their natural hosts.

The analysis revealed that most of the identified insertion sequences were present in multiple copies in *Paracoccus* spp., which most probably reflects the replicative transposition of some of these elements. As shown in Table 2, the highest copy numbers (4–9 copies) were observed for most members of (i) the IS*427* group of the IS*5* family (IS*1248*f, IS*Paes1*, IS*Pbe2*, IS*Pha1*, IS*Ppa2*, IS*Ppa6*, IS*Pze1*), (ii) the IS*903* group of the IS*5* family (IS*Pko1*, IS*Plc1*, IS*Ppa3*a, IS*Ppa8*), (iii) the IS*407* group of the IS*3* family (IS*Pbe1*, IS*Pfe1*), and individual members of (iv) the IS*21* family (IS*Pve1*a) and (v) the IS*66* family (IS*Ppa7*). Some of the detected IS copies displayed different signal intensities in the hybridization analysis, which most probably reflects divergence in the nucleotide sequences of closely related elements or the presence of truncated copies of the same element (data not shown).

In contrast, a low copy number (1–2 copies) was observed for all members of (i) the IS5 group of the IS*5* family (IS*Pha2*, IS*Phae1*, IS*Pmar3*), (ii) the IS*6* family (IS*Pmar1*, IS*Ppa9*) and (iii) the IS*256* family (IS*Paes3*), as well as for the identified transposons (we detected single copies of the non-composite transposons Tn*5393* and Tn*6122*, and of the composite elements, Tn*Ppa1* and Tn*6097*).

Hybridization analysis also revealed that only a few of the TEs identified in this study are located within natural plasmids of *P. aminophilus* JCM 7686, *P. halophilus* JCM 14014, *P. kondratievae* NCIMB 13773^T^, and *P. marcusii* OS22 (Table 2). Thus, the vast majority of the TEs seem to be of chromosomal origin. However, it is worth mentioning that all of the tested paracoccal strains carry megaplasmids, which cannot be purified using a standard alkaline lysis procedure. The question of whether the identified transposable elements reside in these high-molecular weight plasmids remains open.

**Figure 5:**
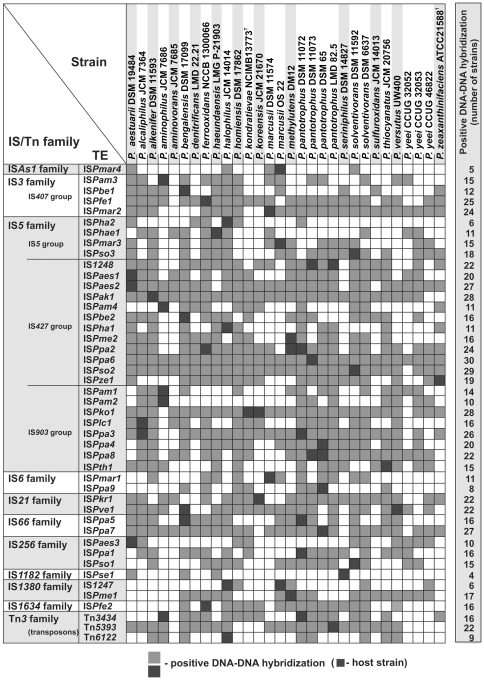
The distribution of TEs (identified in our studies) in the genomes of strains of *Paracoccus* spp. A specific DNA probe (fragment of a TPase gene amplified by PCR and DIG-labeled) was prepared for each TE and used in dot blot hybridization analysis with total DNA isolated from the *Paracoccus* spp. strains.

In the final part of this study we investigated whether the captured TEs are specific only for the host strains, or are widespread among *Paracoccus* spp. (the analysis was extended to all so-far identified TEs of *Paracoccus* spp.; Figure 5). Dot blot hybridization analysis was performed using the DNA probes described above and total DNAs of all tested strains of *Paracoccus* spp. (see Materials and Methods for details). As shown in Figure 5, this analysis revealed that TEs are very widely distributed in *Paracoccus* spp. and that each strain contains several elements from different families. The most common are members of the IS*5* and IS*3* families. In contrast, the elements IS*1182* and IS*As1* appear to be less abundant, which suggests that they may have been acquired by HGT quite recently (Figure 5).

## Discussion

In this study we have identified and analyzed transposable elements of bacteria of the genus *Paracoccus*. For the identification of TEs we used trap plasmids, enabling the positive selection of transposition events. All of the elements were, therefore, defined by their ability to transpose into the selective cassette of these plasmids, which unequivocally confirmed their mobility. This is the first such complex analysis aimed at defining a functional transposable part of the mobilome of a number of strains representing a significant part of one bacterial genus.

The genus *Paracoccus* currently comprises 31 species. In this study we analyzed 25 strains of 20 species isolated from various environments. These represent almost all species of this genus (excluding patented strains) that were available in 2009, when this project started. In total, we tested approximately 7000 individual clones to identify trap plasmid insertion derivatives. However, despite the complexity of this analysis, it is obvious that not all the TEs residing in *Paracoccus* spp. were defined; we only identified those that are the most dynamic in transposition, i.e. elements of highest evolutionary impact. As a result, we captured 41 elements representing (i) insertion sequences, (ii) an IS-driven composite transposon and (iii) non-composite transposons of the Tn*3* family.

In total, together with our previous studies [3], [17]–[20], we have described over 50 novel TEs of *Paracoccus* spp. As expected, the vast majority of the captured elements were insertion sequences. We identified 47 elements, representing 10 IS families: IS*3*, IS*5*, IS*6*, IS*21*, IS*66*, IS*256*, IS*1182*, IS*1380*, IS*1634* and IS*As1*. Interestingly we did not encounter elements of the IS*1*, IS*4*, IS91 or IS110 families, which are widely distributed among other bacteria [46], [47].

The most numerous in *Paracoccus* spp. were members of the IS*5* family (26 elements), while only single elements from the IS*As1*, IS*1182* and IS*1634* families were captured. However, dot blot DNA hybridization revealed that TEs are much more abundant in *Paracoccus* spp. than we had expected. These results suggest that each tested strain carries elements belonging to several different families (Figure 5), the most abundant of which are members of the IS*3*, IS*5*, IS*21*, IS*66* and IS*256* families. In contrast, elements of the IS*As1* and IS*1182* families seem to be rare in *Paracoccus* spp. (Figure 5).

**Figure 6:**
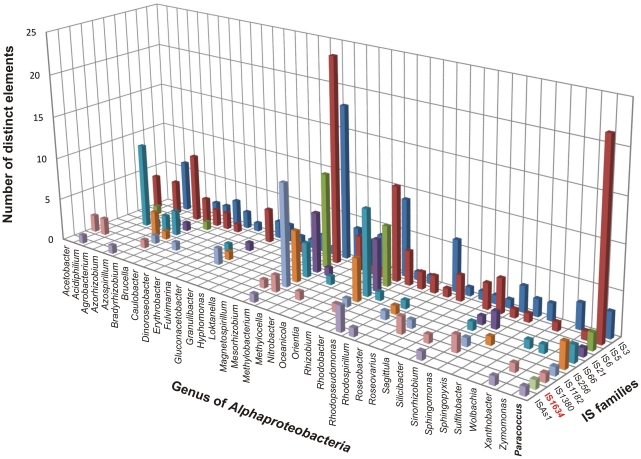
Distribution of IS-families identified in *Paracoccus* spp., among other genera of the class *Alphaproteobacteria* (ISfinder database). The *Paracoccus* spp. elements are members of 10 IS families (IS*3*, IS*5*, IS*6*, IS*21*, IS*66*, IS*256*, IS*1182*, IS*1380*, IS*1634*, IS*As1*). Each IS-family is represented by a different color on the three dimensional histogram. The family IS*1634* unique to the genus *Paracoccus* is shown in red.

The obtained data are consistent with the results of a survey of the ISfinder database to distinguish all ISs identified so far in *Alphaproteobacteria* (Figure 6). This revealed that members of the IS*5* and IS*3* families predominate in this group of bacteria, while ISs of other families are unevenly distributed. In the light of this analysis, *Paracoccus* appears to be the only genus of *Alphaproteobacteria* in which elements representing all 10 of the IS families listed above have been identified (Figure 6). It is also the first genus of this class that has been found to contain an IS*1634* family element, i.e. IS*Pfe2* of *P. ferrooxidans* NCCB 1300066 (Figure 6).

Southern blotting and DNA hybridization analysis revealed that most of the ISs identified in *Paracoccus* spp. are present in multiple copies in their original hosts, and so are potentially able to mobilize different segments of genomic DNA for transposition as composite transposons. In this study we identified one such element, Tn*6097* of *P. ferrooxidans* NCCB 1300066, whose transposition is mediated by two identical copies of IS*Pfe2* (IS*1634* family).

Tn*6097* carries two putative conserved genetic modules. Specific functions of closely related modules have previously been determined in other bacterial hosts. One of the modules carries most probably genes involved resistance to daunorubicin and doxorubicin (used in the treatment of some types of cancer [45]). These chemotherapeutics are active exclusively against gram-positive bacteria, since they do not enter the cells of gram-negative strains [48]. The second module contains putative genes involved in the arginine deiminase (ADI) pathway [45]. The ADI pathway comprises reactions catalyzed by arginine deiminase, ornithine transcarbamylase and carbamate kinase (encoded by the *arcABC* genes), which enable bacteria to grow anaerobically in the presence of arginine. The enzymes mediate the conversion of arginine to ornithine, NH_3_, and CO_2_, with the generation of ATP. It has been shown that this pathway constitutes a major source of energy for several microorganisms, e.g. strains of *Mycoplasma* spp., *Pseudomonas* spp. and *Bacillus* spp. [49].

However, we have observed that *P. ferrooxidans* NCCB 1300066 (host strain of Tn*6097*) is unable to grow anaerobically in the presence of arginine (data not shown), which suggests that the *arcABCD* operon may be not functional. It has been previously demonstrated that anaerobic expression of the closely related operon of *Pseudomonas aeruginosa*, requires a transcriptional activator [50], which is not encoded within Tn*6097*. It is therefore possible that the ADI module of Tn*6097* may be functional exclusively in the strains encoding a compatible transcription regulator.

We have also identified several non-composite TEs of the Tn*3* family, which represent different stages in the evolution of this group of transposons. It is believed that the Tn*3* transposons originate from progenitor IS elements (several such ISs coding for the Tn*3*-like transposase have been identified; ISfinder), which acquired an additional genetic module involved in site-specific resolution of cointegrates (intermediates of replicative transposition) as well as other genes whose products can directly affect the phenotype of their host [51].

In *Paracoccus* spp. we identified two cryptic transposons (Tn*6122* and Tn*3434*a) that contain the conserved module for site-specific recombination. We also captured the transposon Tn*5393* (closely related to Tn*3434*a), which apart from the resolvase module, carries two additional streptomycin resistance genes (Figure 2). Interestingly, analysis of the G+C profiles of these transposon nucleotide sequences revealed that the individual genetic modules of the elements are bordered by short stretches of AT-rich sequence (Figure 2), which presumably constitute traces of consecutive recombinational events that led to incorporation of the different portions of genetic information into the transposons.

The next step in the evolution of Tn*3* elements is represented by Tn*Ppa1* (44,286 bp) of *P. pantotrophus* DSM 11072, which can be considered a composite transposon generated by two copies of the non-composite Tn*3434* [18]. The identification of Tn*Ppa1* provides evidence that the Tn*3*-family transposons (similarly to ISs) are able to mobilize large segments of genomic DNA for transposition. The generation of such composite elements seems to be very rare because of transposition immunity (a phenomenon that is thought to apply to members of the Tn*3* family), which precludes transposition of more than one copy of the element into a single replicon [11]. Therefore Tn*Ppa1*, composed of two identical copies of Tn*3434*, has a unique structure and, to our knowledge, is the only element of this type identified so far.

In this study, we found that Tn*Ppa1* is able to transpose into random sites of several mobilizable plasmids with unusually high frequency, not previously observed for any other bacterial TE. Our observations strongly suggest that the massive transposition of Tn*Ppa1* might result from the specific features of the transferred plasmids. Irrespective of the precise mechanism of Tn*Ppa1* transposition, it is clear that targeting of mobilizable or conjugal plasmids contributes to the dispersal of this element among bacterial populations. Within its core region, Tn*Ppa1* carries a number of genes (encoding putative enzymes involved in central intermediary metabolism, membrane transporters, transcription regulators and proteins of unknown function), whose presence may potentially improve the ecological fitness of the host cells [18]. Transposition of such a large portion of genetic information (containing putative housekeeping genes) into co-residing plasmids significantly enriches the pool of mobile DNA, which may then be spread by HGT among even phylogenetically-distinct bacterial hosts, to produce a variety of phenotypic effects [18]. The transposition of TEs containing housekeeping genes into bacterial plasmids may also have a significant impact on the structure and evolution of bacterial genomes, e.g. it may stimulate the formation of multi-chromosome genomes, which are very common in *Alphaproteobacteria* (the presence of two chromosomes has also been demonstrated for two paracoccal strains, whose genomes have been fully sequenced) [52], [53].

In this study, we also observed very frequent transposition (10^−3^) of another member of the Tn*3* family: the streptomycin resistance Tn*5393* (*P. pantotrophus* LMD 82.5) – far higher than that observed for other non-composite members of this family (Table S3) (e.g. the transposition frequency of Tn*3434* was 10^−6^) [17]. Such dynamic transposition of Tn*5393* explains the wide dissemination of this transposon in many bacterial isolates [54], [55] and is evidence that transposition may be a key mechanism responsible for the natural amplification of antibiotic resistance genes in environmental bacterial strains.

We have found that the Tn*5393* TPase gene carries a strong promoter, which is able to drive the transcription of genes placed downstream of the target sites of transposition. It is therefore highly probable that the frequent transposition of Tn*5393* is directly linked with the strength of this promoter.

Using the trap plasmid pCM132TC containing a promoterless tetracycline resistance gene, we identified a pool of TEs whose promoters are able to drive transcription of genes placed downstream of the target site of transposition. Such elements therefore represent portable expression systems that enable activation of single promoterless genes or operons as well as foreign genes introduced by HGT whose native promoters are non-functional in their new hosts. Thus transposition of these elements may result in the immediate appearance of novel phenotypes of adaptative value [13].

We showed that activation of nearby genes is a typical feature of insertion sequences of the IS*3*, IS*21* and IS*256* families, which corroborates the findings of previous studies [56], [57]. Moreover, for the first time, we have demonstrated such ability for (i) elements of the IS*903* group of the IS*5* family (contain an outwardly oriented promoter in the terminal part of the elements), and (ii) a transposon of the Tn*3* family (the aforementioned Tn*5393*).

The individual transposition events which led to the Tc^r^ phenotype (activation of the promoterless *tetA* gene of pCM132TC) were examined in detail. This analysis revealed that transposition-mediated delivery of promoters to transcriptionally silent genes can occur in at least five ways (Figure 3). The most intriguing was observation that transcription can be driven by promoters derived from natural plasmids, which form transient co-integrates with other co-residing replicons as a result of replicative transposition. We observed that such co-integrates can be quite stably maintained even in the absence of selective pressure (tetracycline), which supports their role as efficient expression systems.

Another interesting phenomenon observed in this study was connected with the insertion sequence IS*Pve1* (IS*21* family), which was prone to generate deletions within target DNA molecules (Figure 4). The transposition in a cell of IS*Pve1* therefore results in the generation of a mixed population of plasmid molecules. The resulting diminished forms of the replicons may potentially differ in their specific properties (e.g. stability and copy number). This indicates that transposition may significantly shape plasmid genomes and promote plasmid diversity, which could have significant evolutionary implications.

Interestingly, this “deletion” phenomenon was not observed for other members of the IS*21* family identified in this study, including IS*Pve1*a, an isoform of IS*Pve1* from *P. bengalensis* DSM 17099 (99% identity at the nucleotide sequence level). The high frequency of deletions generated by IS*Pve1* (in comparison with “pure” insertion events) might therefore be a specific feature of this element. Alternatively, it might result from the presence of host factors, which stimulate such deletions. The latter hypothesis is supported by data recently published by Kusumoto *et al.* [58], which provide evidence that deletions of ISs or DNA regions adjacent to them might be promoted (in an IS transposase-dependent manner) by a chromosomally-encoded protein IEE (IS-excision enhancer). Further studies on IS*Pve1* and the strain *P. versutus* UW400 are necessary to determine the likelihood of these two possibilities.

To conclude, the analysis performed in this study together with our previous work concerning the identification of diverse TMos generated by a single copy of IS*Pme1* [3], have highlighted the diversity and wide distribution of TEs in *Paracoccus* spp. It should also be borne in mind that many TEs (including composite transposons and TMos) cannot be distinguished in bacterial genomes by classical *in silico* sequence analysis, unless they have been inserted into a highly conserved genetic context (e.g. a trap plasmid selection cassette), to enable precise definition of their termini. For this reason, the identification of functional TEs using trap plasmids may produce many interesting and surprising findings even when carried out in bacteria whose genomes have been fully sequenced.

Our analysis of the transposable part of the mobilome of *Paracoccus* spp. has also generated considerable interesting and unique data concerning the dynamics of the process of transposition (Table S3). The results also indicate the powerful role of transposition in the dissemination of diverse genetic information (possibly of adaptative value) by HGT. Considering their status as one of the most recombinogenic factors in bacterial genomes, TEs can be considered as the major driving force in the evolution of prokaryotes.

## Supporting Information

Figure S1 **Multiple alignment of nucleotide sequences of the terminal inverted repeats (IRL, IRR) of IS-families identified in** ***Paracoccus*** **spp. (IS** ***3*** **, IS** ***5*** **, IS** ***6*** **, IS** ***21*** **, IS** ***66*** **, IS** ***256*** **, IS** ***1182*** **, IS** ***1380*** **, IS** ***1634*** **, IS** ***As1*** **).** The sequences used for the comparisons were obtained from the ISfinder database (http://www-is.biotoul.fr/is.html). Sequence alignments were performed with the Pictogram program (WEBLOGO; http://weblogo.berkeley.edu/logo.cgi). The size of the letter in Pictogram represents the frequency of the occurrence of that nucleotide at each position. In the consensus sequences, shown below each Pictogram, uppercase letters indicate conservation within the IS family, lowercase letters indicate the predominant nucleotides, and dots indicate non-conserved residues. The family and group specific DDE motifs determined by multiple alignment of the amino acid sequences of the transposases of ISs present in the ISfinder database has been shown in the middle of the figure ([5] and this study). Residues forming the DDE motif are indicated with a black background. The N2, N3 and C1 domains are enclosed in boxes and labeled. The numbers in parentheses are the distances (in amino acids – aa) between the residues forming the DDE motif. The consensus IR and DDE motif sequences for each IS-family is compared with all sequences identified so far in *Paracoccus* spp.
(TIF)

Table S1 **Oligonucleotides used in this study.**
(DOC)

Table S2 **ORFs located within Tn** ***6097*** **of** ***Paracoccus ferrooxidans*** **NCCB 1300066.**
(DOC)

Table S3 **Rate of transposition of the TEs of** ***Paracoccus*** **spp. identified in this study.**
(DOCX)
