# Supplementary figures and images for: Insights into the Transposable Mobilome of Paracoccus spp. (Alphaproteobacteria)
Source: PLoS One. 2012 Feb 16;7(2):e32277. doi: 10.1371/journal.pone.0032277 (PMC3281130; doi:10.1371/journal.pone.0032277)

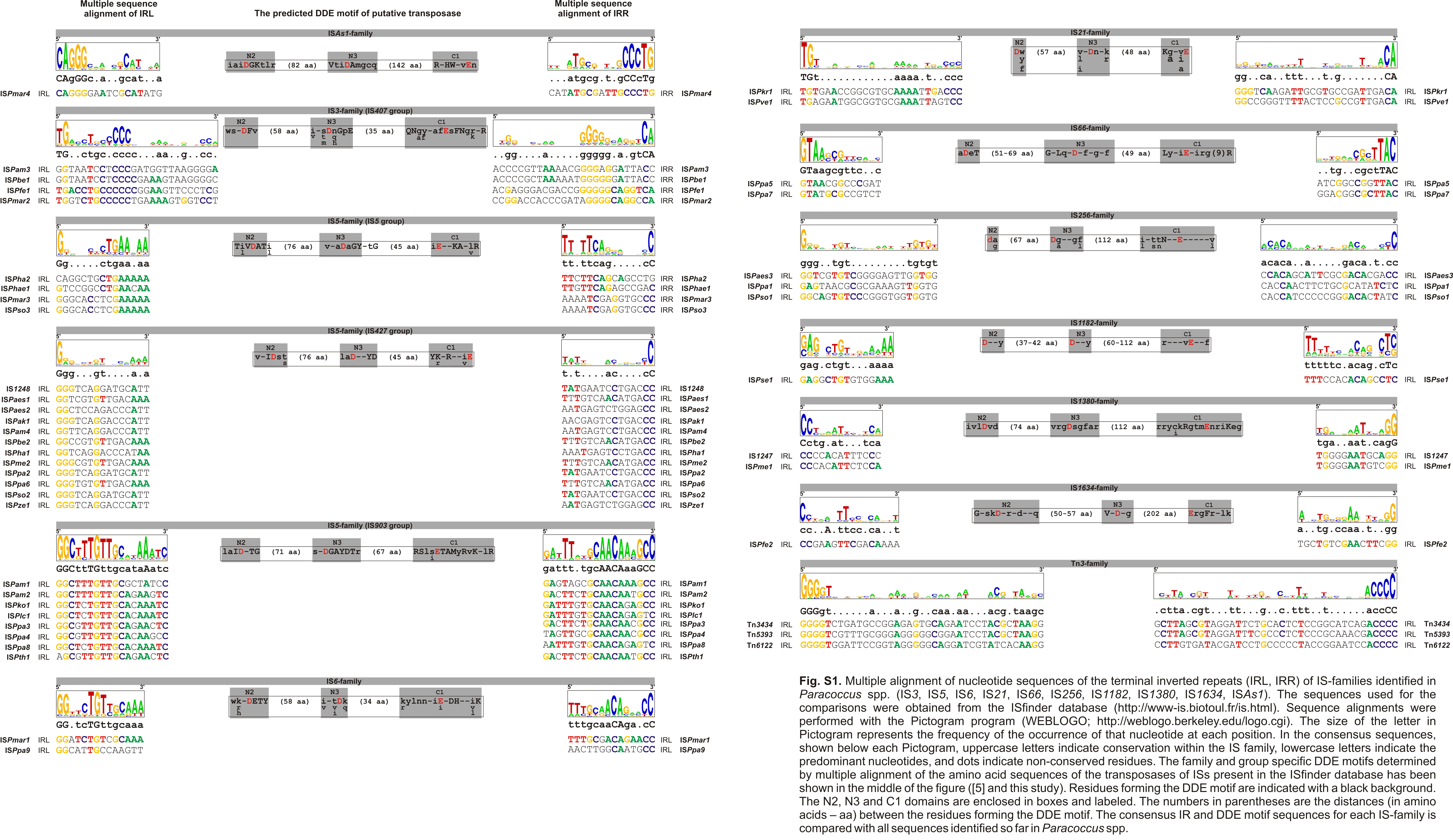

Supplement: Figure S1 — Multiple alignment of nucleotide sequences of the terminal inverted repeats (IRL, IRR) of IS-families identified in Paracoccus spp. (IS 3 , IS 5 , IS 6 , IS 21 , IS 66 , IS 256 , IS 1182 , IS 1380 , IS 1634 , IS As1 ). The sequences used for the comparisons were obtained from the ISfinder database (http://www-is.biotoul.fr/is.html). Sequence alignments were performed with the Pictogram program (WEBLOGO; http://weblogo.berkeley.edu/logo.cgi). The size of the letter in Pictogram represents the frequency of the occurrence of that nucleotide at each position. In the consensus sequences, shown below each Pictogram, uppercase letters indicate conservation within the IS family, lowercase letters indicate the predominant nucleotides, and dots indicate non-conserved residues. The family and group specific DDE motifs determined by multiple alignment of the amino acid sequences of the transposases of ISs present in the ISfinder database has been shown in the middle of the figure ([5] and this study). Residues forming the DDE motif are indicated with a black background. The N2, N3 and C1 domains are enclosed in boxes and labeled. The numbers in parentheses are the distances (in amino acids – aa) between the residues forming the DDE motif. The consensus IR and DDE motif sequences for each IS-family is compared with all sequences identified so far in Paracoccus spp. (TIF) [file pone.0032277.s001.tif]
