# Supplementary material for: Insights into the Transposable Mobilome of Paracoccus spp. (Alphaproteobacteria)
Source: PLoS One. 2012 Feb 16;7(2):e32277. doi: 10.1371/journal.pone.0032277 (PMC3281130; doi:10.1371/journal.pone.0032277)
Supplement: Table S1 — Oligonucleotides used in this study. (DOC) [file pone.0032277.s002.doc]

| **Primers used for the identification of TEs**  **Table S1.** Oligonucleotides used in this study. | | | | | |
| --- | --- | --- | --- | --- | --- |
| **Trap vector** | **Primer** | **Sequence (5’→3’)** | **Trap vector** | **Primer** | **Sequence (5’→3’)** |
| **pMEC1/**  **pMMB2** | ALIS | TTGTAATCAGCTATGCGCCG | **pMAT1/**  **pEBB10** | A489SB | CAGACCGCTAACACAGTACA |
|  | ARIS | TCTGGCTTGAGGTTGAAGGT |  | A869SB | TTAGGATCTCCGGCTAATGC |
|  | BLIS | TGGTGCGGTCATGGAATTAC |  | B824SB | ACTATCACGGCTACCACATC |
|  | BRIS | GTATGCAGCCGTCACTTAGA |  | B1253SB | TTGTCGCCTGAGCTGTAGTT |
|  | CLIS | TCCCTGCCTGAACATGAGAA |  | C1225SB | GATGAAGGCAACTACAGCTC |
|  | CRIS | ACACAAGAGCAGCTTGAGGA |  | C1639SB | ACGTAATGCCGTCAATCGTC |
|  | DLIS | TCTTGTCTGCGACAGATTCC |  | D1619SB | TGACGATTGACGGCATTACG |
|  | DRIS | TTCATACACGGTGCCTGACT |  | D1928SB | CCTTGTTCAAGGATGCTGTC |
|  | ELIS | GGTTGCATGTACTAAGGAGG | **pCM132TC** | RTETCM | GCAAGACTGGCATGATAAGG |
|  | ERIS | GCAAGACTGGCATGATAAGG |  |  |  |
| **Primer pairs used for the PCR amplification of hybridization probes** | | | | | |
| **TE** | **Primer** | **Sequence (5’→3’)** | **TE** | **Primer** | **Sequence (5’→3’)** |
| **IS*1247*/**  **IS*1247a*** | LTNP2TMo | GTGATGCGCGAGCTTGATGA | **IS*Pmar4*** | LTNP1TMo | GAACACGCGACATGACTTGG |
|  | RTNP2TMo | GAAGAACCGCTTGACCTTGG |  | RTNP1TMo | AGGACAGCGCGAAGTACCTC |
| **IS*1248*/**  **IS*1248f*** | LSIS1248 | GACATGAGCGACCTGTACTG | **IS*Pme1*** | LSISPME1 | ATCATGGCGTTGACGGCTGC |
|  | RSIS1248 | GATGGCCGAGAGGAAGACCT |  | RSISPME1 | ACTGGAAGGTAATGGCGCGG |
| **IS*Paes1*** | LSISPAE1 | TGGCAGTCGTCGTATGATTC | **IS*Pme2*** | LSISPME2 | CCACGAACCATCGGCTTGTT |
|  | RSISPAE1 | GTTGCGGCTCTCGATGTCTT |  | RSISPME2 | TGGTCTTGCGGATGTTGTCG |
| **IS*Paes2*** | LSISPAE2 | TCCTCAAGGAGACCTGATCG | **IS*Ppa1*** | MEC114L | TTCTGACCGTCGAGCTTGCG |
|  | RSISPAE2 | CATGATCGCTGAGAAGAACG |  | MEC114R | GCGGCATATCAAGGCGGTGT |
| **IS*Paes3*** | LSISPAE3 | GCCATGAACCAGATTACCGA | **IS*Ppa2*** | MEC1248L | TGTACTGGCTGACCAACGAG |
|  | RSISPAE3 | AGCCGTGTGAAGGTGAAGAG |  | MEC1248R | TGGCTGAGAGGAACACCTTG |
| **IS*Pak1*** | LSISPAK1 | TCGATGACCGGCGTGTTCTC | **IS*Ppa3*** | MEC181L | ATGAGATGCGTTCACCGAAG |
|  | RSISPAK1 | AACATGACGGTTGCGGCGAG |  | MEC181R | TGGAAGTCCTACAACGATGC |
| **IS*Pam1*** | LISPam1S | GGCGTGCCGAGTGAAGTGAA | **IS*Ppa4*** | LSISPPA4 | TGCATGAGCAAGCCATCACC |
|  | RISPam1S | CCGTATGAGCAAGCCTGTCC |  | RSISPPA4 | AATCTTCCTTCCACAGCCGC |
| **IS*Pam2*** | LSISPAM2 | CCTGCTGATCTGGTTGGACA | **IS*Ppa5*/**  **IS*Ppa5a*** | LSISPPA5 | GGTATAGACGCTGATGATCG |
|  | RSISPAM2 | GAGTGCTACGCGGATCTGGA |  | RSISPPA5 | GGCACCAACTGAAGAAGAAG |
| **IS*Pam3*** | LPAM3S | GGCGTTCGTTGTTGTAGGTC | **IS*Ppa6*** | LISPPA6 | CCTGATGTCCGACGACGAAT |
|  | RPAM3S | ACAGATCATGCGCGTGCTGC |  | RISPPA6 | AAGCGGCGACTGTTCTTGAG |
| **IS*Pam4*** | LSISPAM4 | GGTTGCGTGATTCAAGCTCC | **IS*Ppa7*** | LISPPA7 | CGCCAAGCATTCTGAACACA |
|  | RSISPAM4 | CAGAAGATGACGGTTGCAGC |  | RISPPA7 | GCATCAAGGCGTCTATGTCC |
| **IS*Pbe1*** | LISPBE1 | AAGTCCAGAGCCATTGCGTG | **IS*Ppa8*** | LISPPA8 | CCGCATACAAGACCAGGAAC |
|  | RISPBE1 | AATGACGCGGCCATCTCAAC |  | RISPPA8 | TTCAGGACGGCGACACGGAT |
| **IS*Pbe2*** | LSISPBE2 | CTTCATTCGCGCCGTCAGAC | **IS*Ppa9*** | LISPPA9 | ATCATTGCCTACGCCGTCTG |
|  | RSISPBE2 | GAAGCAACGCTCGACCATGT |  | RISPPA9 | GTGCGTGGCGGTATGAAGAT |
| **IS*Pfe1*** | LSISPFE1 | ACGTTGTTGTAGTCGTAGCG | **IS*Pse1*** | LSISPSE1 | CCGAGCGTCTCGAAGATTGG |
|  | RSISPFE1 | GCTGTGATGTAGAGTCTGCC |  | RSISPSE1 | GCGACCTCCGACCATTCCTT |
| **IS*Pfe2*** | LSISPFE2 | GGACGGCTCTTAGGCAGGTT | **IS*Pso1*** | LSISPSO1 | GCCGATCTTCGGAGAAGGTT |
|  | RSISPFE2 | GCTCGTCGAGTCCTATCGGA |  | RSISPSO1 | GGAAGGTCATGTACGCCAGC |
| **IS*Pha1*** | ISPha1L | GGCAACATGAGCGACTACTA | **IS*Pso2*** | LSISPSO2 | ACATGAGCGATCTCTTCTGG |
|  | ISPha1R | AGGTCTCCGCATCTGTCGTA |  | RSISPSO2 | CCGCTTGTATCTCCGCTTAT |
| **IS*Pha2*** | ISPha2L | CGTGAACGATGCACTGACGA | **IS*Pso3*** | LSISPSO3 | GAGATTGACGCCGTTGTGCC |
|  | ISPha2R | AAGAAGTCGGCCTTGAAGGA |  | RSISPSO3 | GCGCATGTTGTAAGCCAGGT |
| **IS*Phae1*** | ISMABCD | CAACACTTCACTGGCGAGAC | **IS*Pth1*** | LSISPTH1 | GGTTAAGAGCCTGCCATGAG |
|  | RISPHA1 | GTCAGGCGGCTAATACTTCC |  | RSISPTH1 | ATGTGGATCTCGGCTGTCTG |
| **IS*Pko1*/**  **IS*Pko1a*** | LSISPKO1 | CTACAGGACCAGGAACTGGC | **IS*Pve1*/**  **IS*Pve1a*** | LSISPVE1 | GGAGGATCTACAGCGTGGAA |
|  | RSISPKO1 | TTCAGAACGGCGATACGGAC |  | RSISPVE1 | TTCTCGCAGGAAGGTCGGCA |
| **IS*Pkr1*** | LSISPKR1 | GAGATATTGGCGGCGAACTC | **IS*Pze1*** | ISPZEAL | CTGGCTGACGGACGAACAGA |
|  | RSISPKR1 | TTCGACGATGTCGCAGTGAT |  | ISPZEAR | ATCGTAACGTGTCGCTACTC |
| **IS*Plc1*** | LSISPLC1 | TGTTGTTCGGCATGGCCTTG | **Tn*3434*/**  **Tn*3434a*** | L1TN3434 | CGTGCGTTCGCGGATCAACT |
|  | RSISPLC1 | CGCGGTGTAACCGTTCAGGA |  | R1TN3434 | TCGACAGCGTGACCGAGATG |
| **IS*Pmar1*** | LSISPMA1 | TGAGGATTGTGCAATGAGCG | **Tn*5393*** | LTN5393 | CCAGCGATATTCTCCGGTGA |
|  | RSISPMA1 | GATCTGACGGAACGTCTGAA |  | RTN5393 | GGCTTGCGATCTTGGCTGTT |
| **IS*Pmar2*** | LSISPMA2 | TTGGAGGATGCAGGAATGCC | **Tn*6122*** | MEH55FH | ATGATCGACCGTCTTCCACG |
|  | RSISPMA2 | CGTCGCGGAGCTTGGAATTG |  | MEH55RH | GGCCAGTTCTGGACGATGAC |
| **IS*Pmar3*** | ISPmar3L | TGCCGTGCTGATGTTCAAGA |  |  |  |
|  | ISPmar3R | CCTCCTGGCCTGATTACCGT |  |  |  |
| **Primers used for the determination of the sequences flanking transposon Tn*6097* (IPCR)** | | | | | |
| **TE** | **Primer** | **Sequence (5’→3’)** |  |  |  |
| **Tn*6097*** | LIPCRPF2 | CGAAGAGTTCCGCCTCAGCA |  |  |  |
|  | RIPCRPFE | CCTCGTTCTCTACCGCGTCA |  |  |  |
| **Primers used for the identification of promoters of TEs** | | | | | |
| **TE** | **Primer** | **Sequence (5’→3’)** | **TE** | **Primer** | **Sequence (5’→3’)** |
| **IS*Pam1*** | LP2PAM1 | cgaattcAGTCGCGTCGAGGCCAAGAT | **IS*Paes3*** | LPAEFUZ | **cgaattc**ACAAGCACCGCAACCTCCTG |
|  | RP2PAM1 | tggatccGGCTTTGTTGCGCTACTCGG |  | RPAEFUZ1 | **tggatcc**GGTCGTGTCGCGAATGCTGT |
| **IS*Pkr1*** | PKR1L | **cgaattc**GGTCGTCATCAACATCCTGG |  | RPAEFUZ2 | **tggatcc**CAAGACTGGCATGATAAGGC |
|  | PKR1PIR | **tggatcc**TGTCAATCGGCACGCAATCT |  |  |  |
|  | PKR1Pkas | **tggatcc**AGCGCGGATCATCGGTCAGA |  |  |  |
