# Supplementary material for: Insights into the Transposable Mobilome of Paracoccus spp. (Alphaproteobacteria)
Source: PLoS One. 2012 Feb 16;7(2):e32277. doi: 10.1371/journal.pone.0032277 (PMC3281130; doi:10.1371/journal.pone.0032277)
Supplement: Table S2 — ORFs located within Tn 6097 of Paracoccus ferrooxidans NCCB 1300066. (DOC) [file pone.0032277.s003.doc]

**Table S2.** ORFs located within Tn*6097* of *Paracoccus ferrooxidans* NCCB 1300066.

| ORF no. | **Coding region (bp)** | **Orientation** | **Protein size (aa)** | **Possible function** | **Best BLAST hits** | | |
| --- | --- | --- | --- | --- | --- | --- | --- |
|  |  |  |  |  | **Percentage identity (aa)** | **Organism** | **GenBank accession no.** |
| 1 | 80-1726 | ← | 548 | Putative transposase | 81  (445/548) | *Rhodobacter sphaeroides* ATCC  17025 | YP_001167631 |
| 2 | 1885-2415 | → | 176 | Putative truncated transcriptional activator | 75  (131/175) | *Roseobacter denitrificans* OCh  114 | YP_682690 |
| 3 | 2711-3916 | ← | 401 | Putative uncharacterized protein involved in  response to NO | 71  (284/401) | *Roseovarius* sp. TM1035 | ZP_01879375 |
| 4 | 3913-4302 | ← | 129 | Hypothetical protein of  5-formyltetrahydrofolate cyclo-ligase family | 56  (68/122) | *Roseovarius* sp. TM1035 | ZP_01035279 |
| 5 | 4306-4686 | ← | 126 | Hypothetical protein of Regulator of cell morphogenesis and NO signaling | 54  (42/78) | *Roseovarius* sp. TM1035 | ZP_01036683 |
| 6 | 4926-5843 | ← | 305 | Carbamate kinase; ArcC-like protein | 72  (218/302) | *Ochrobactrum anthropi* ATCC 49188;  plasmid pOANT03 | YP_001373248 |
| 7 | 5848-6849 | ← | 333 | Ornithine carbamoyltransferase; ArcB-like protein | 84 (279/331) | *Ochrobactrum anthropi* ATCC 49188;  plasmid pOANT03 | YP_001373247 |
| 8 | 6863-8089 | ← | 408 | Arginine deiminase; ArcA-like protein | 85 (347/407) | *Ochrobactrum anthropi* ATCC 49188;  plasmid pOANT03 | YP_001373246 |
| 9 | 8105-9538 | ← | 477 | C4-dicarboxylate anaerobic carrier; ArcD-like protein | 81 (384/476) | *Ochrobactrum anthropi* ATCC 49188;  plasmid pOANT03 | YP_001373245 |
| 10 | 10236-11321 | → | 361 | Putative secretion protein of HlyD family | 56 (194/348) | *Ochrobactrum anthropi* ATCC 49188 | YP_001370368 |
| 11 | 11284-14086 | → | 933 | Putative ABC transporter protein; DrrA-like protein | 67  (620/932) | *Xanthobacter autotrophicus* Py2;  plasmid pXAUT01 | YP_001409357 |
| 12 | 14090-15202 | → | 370 | Putative ABC-2 type transporter protein; DrrB-like protein | 65  (240/370) | *Xanthobacter autotrophicus* Py2;  plasmid pXAUT01 | YP_001409356 |
| 13 | 15485-15875 | ← | 129 | Putative truncated NAD-dependent epimerase/  dehydratase | 85 (110/129) | *Paracoccus denitrificans* PD1222;  plasmid 1 | YP_918330 |
| 14 | 15955-17601 | ← | 548 | Putative transposase | 81  (445/548) | *Rhodobacter sphaeroides* ATCC  17025 | YP_001167631 |
