# Supplementary material for: Insights into the Transposable Mobilome of Paracoccus spp. (Alphaproteobacteria)
Source: PLoS One. 2012 Feb 16;7(2):e32277. doi: 10.1371/journal.pone.0032277 (PMC3281130; doi:10.1371/journal.pone.0032277)
Supplement: Table S3 — Rate of transposition of the TEs of Paracoccus spp. identified in this study. (DOCX) [file pone.0032277.s004.docx]

**Table S3.** Rate of transposition of the TE of *Paracoccus* spp. identified in this study.

| **TE (family/ group)** | **Host strain** | **Trap**  **plasmid** | **Trap plasmid mutants containing the element [%]** | **Frequency of transposition** |
| --- | --- | --- | --- | --- |
| **IS*1247*** (IS*1380*) | *P. halophilus* JCM14014 | pMEC1 | 5 | 1.2 ×10^-5^ |
| **IS*1248*** (IS*5*/IS*427*) | *P. pantotrophus* DSM 11073 | pCM132TC | 82 | 1.1 × 10^-6^ |
| **IS*1248f*** (IS*5*/IS*427*) | *P. pantotrophus* LMD 82.5 | pMAT1 | 5 | 6.0 × 10^-5^ |
| **IS*Paes1*** (IS*5*/IS*427*) | *P. aestuarii* DSM 19484 | pMEC1 | 5 | 1.0 × 10^-6^ |
| **IS*Paes2*** (IS*5*/IS*427*) | *P. aestuarii* DSM 19484 | pMAT1 | 5 | 1.5 × 10^-5^ |
| **IS*Paes3*** (IS*256*) | *P. aestuarii* DSM 19484 | pCM132TC | 50 | 3.6 × 10^-10^ |
| **IS*Pak1*** (IS*5*/IS*427*) | *P. alkenifer* DSM 11593 | pMAT1 | 71 | 0.8 × 10^-4^ |
| **IS*Pam1*** (IS*5*/IS*903*) | *P. aminophilus* JCM 7686 | pEBB10 pCM132TC | 39  20 | 5.0 × 10^-7^  2.2 × 10^-4^ |
| **IS*Pam2*** (IS*5*/IS*903*) | *P. aminophilus* JCM 7686 | pEBB10 | 16 | 2.1 × 10^-7^ |
| **IS*Pam3*** (IS*3*/IS*407*) | *P. aminophilus* JCM 7686 | pCM132TC | 28 | 3.1 × 10^-4^ |
| **IS*Pbe1*** (IS*3*/IS*407*) | *P. bengalensis* DSM 17099 | pMEC1 | 6 | 1.6 × 10^-7^ |
| **IS*Pbe2*** (IS*5*/IS*427*) | *P. bengalensis* DSM 17099 | **pMEC1** | 3 | 8.0 × 10^-8^ |
| **IS*Pfe1*** (IS*3*/IS*407*) | ***P. ferrooxidans* NCCB 1300066** | **pCM132TC** | 21 | 5.5 × 10^-8^ |
| **IS*Pfe2*** (IS*1634*) | ***P. ferrooxidans* NCCB 1300066** | pMAT1 | 8 | 1.6 × 10^-5^ |
| **IS*Pha1*** (IS*5*/IS*427*) | *P. halophilus* JCM14014 | pMEC1 | 12 | 2.9 × 10^-5^ |
| **IS*Pha2*** (IS*5*/IS*5*) | *P. halophilus* JCM14014 | pMEC1 | 5 | 1.2 × 10^-5^ |
| **IS*Phae1*** (IS*5*/IS*5*) | *P. haeundaensis* LMG P-21903 | pMAT1 | 31 | 1.8 × 10^-5^ |
| **IS*Pko1*** (IS*5*/IS*903*) | *P. kondratievae* NCIMB 13773T | pMEC1 pCM132TC | 46  31 | 4.4 × 10^-6^  3.4 × 10^-9^ |
| **IS*Pko1a*** (IS*5*/IS*903*) | *P. koreensis* JCM 21670 | pMEC1  pMMB2 | 99  96 | 1.7 × 10^-6^  9.6 × 10^-6^ |
| **IS*Pkr1*** (IS*21*) | *P. koreensis* JCM 21670 | pCM132TC | 38 | 1.8 × 10^-7^ |
| **IS*Plc1*** (IS*5*/IS*903*) | *P. alcaliphilus* JCM 7364 | pMAT1 | 25 | 1.4 × 10^-5^ |
| **IS*Pmar1*** (IS*6*) | *P. marcusii* DSM 11574 | pMMB2 | 6 | 3.0 × 10^-7^ |
| **IS*Pmar2*** (IS*3*/IS*407*) | *P. marcusii* DSM 11574 | pMEC1 | 3 | 3.3 × 10^-7^ |
| **IS*Pmar3*** (IS*5*/IS*5*) | *P. marcusii* OS22 | pMAT1  pMEC1  pCM132TC | 94  90  40 | 6.7 × 10^-5^  1.1 × 10^-4^  7.2 × 10^-7^ |
| **IS*Ppa2*** (IS*5*/IS*427*) | *P. ferrooxidans* NCCB 1300066 | pMAT1 | 4 | 4.0 × 10^-6^ |
| **IS*Ppa3a*** (IS*5*/IS*903*) | *P. alcaliphilus* JCM 7364 | pMAT1 | 38 | 2.1 × 10^-5^ |
| **IS*Ppa5a*** (IS*66*) | *P. bengalensis* DSM 17099 | pMEC1 | 2 | 5.0 × 10^-8^ |
| **IS*Ppa6*** (IS*5*/IS*427*) | *P. pantotrophus* DSM 11073 | pMEC1 | 100 | 1.1 × 10^-3^ |
| **IS*Ppa7*** (IS*66*) | *P. pantotrophus* DSM 65 | pMEC1 | 16 | 1.4 × 10^-5^ |
| **IS*Ppa8*** (IS*5*/IS*903*) | *P. pantotrophus* DSM 65  *P. pantotrophus* DSM 11073 | pCM132TC | 8  8 | 1.4 × 10^-8^  1.1 × 10^-7^ |
| **IS*Pse1*** (IS*1182*) | *P. seriniphilus* DSM 14827 | pMAT1 | 18 | 1.1 × 10^-4^ |
| **IS*Pth1*** (IS*5*/IS*903*) | *P. thiocyanatus* JCM 20756 | pMAT1 | 100 | 1.5 × 10^-5^ |
| **IS*Pve1*** (IS*21*) | *P. versutus* UW400 | pMEC1 | 8 | 2.1 × 10^-7^ |
| **IS*Pve1*a** (IS*21*) | *P. bengalensis* DSM 17099 | pMEC1 | 7 | 1.9 × 10^-7^ |
| **IS*Pze1*** (IS*5*/IS*427*) | *P. zeaxanthinifaciens* ATCC 21588^T^ | pMEC1 | 40 | 3.6 × 10^-3^ |
| **Tn*3434*a** (Tn*3*) | *P. aminophilus* JCM 7686 | pEBB10 | 27 | 3.5 × 10^-7^ |
| **Tn*5393*** (Tn*3*) | *P. pantotrophus* LMD 82.5 | pMAT1  pMEC1  pCM132TC | 94  100  100 | 1.1 × 10^-3^  1.2 × 10^-3^  3.7 × 10^-5^ |
| **Tn*6097*** | *P. ferrooxidans* NCCB 1300066 | pCM132TC | 1 | 3.0 × 10^-10^ |
| **Tn*6122*** (Tn*3*) | *P. halophilus* JCM14014 | pMEC1 | 75 | 1.8 × 10^-4^ |
| **Tn*Ppa1*** (Tn*3*) | *P. pantotrophus* DSM 11072 | pMMB2, pCM132TC | 10  2 | 2.1 × 10^-7^  3.4 × 10^-9^ |
